# Plasma Lipidomic Phenotype Associated with Pathogenic *LDLR* Variants in Familial Hypercholesterolemia

**DOI:** 10.3390/ijms27209011

**Published:** 2026-10-11

**Authors:** Joanna Waś, Aleksander Prejbisz, Magdalena Niedolistek, Ilona Kowalik, Ilona Minota, Joanna Ponińska, Anna Drohomirecka, Dorota Sokołowska, Rafał Płoski, Piotr Dobrowolski

**Affiliations:** 1 Department of Medical Biology, National Institute of Cardiology, State Research Institute, 04-628 Warsaw, Poland; mniedolistek@ikard.pl (M.N.); iminota@ikard.pl (I.M.); dsokolowska@ikard.pl (D.S.); rploski@wp.pl (R.P.); 2 Department of Epidemiology, Cardiovascular Disease Prevention and Health Promotion, National Institute of Cardiology, State Research Institute, 04-628 Warsaw, Poland; aprejbisz@ikard.pl (A.P.); pdobrowolski@ikard.pl (P.D.); 3 Clinical Research Support Centre, National Institute of Cardiology—State Research Institute, 04-628 Warsaw, Poland; ikowalik@ikard.pl; 4 Heart Failure and Transplantology Clinic, National Institute of Cardiology—State Research Institute, 04-628 Warsaw, Poland; adrohomirecka@ikard.pl; 5 Department of Medical Genetics, Medical University of Warsaw, 02-106 Warsaw, Poland

**Keywords:** cardiovascular high risk (HCVR), lipidomics, metabolomics, familial hypercholesterolemia, pathogenic variants, *LDLR*

## Abstract

Familial hypercholesterolemia (FH) is caused by an impaired ability to clear circulating low-density lipoprotein (LDL-C) particles due to a defective low-density lipoprotein receptor (*LDLR*). FH significantly increases cardiovascular risk (CVR). Although conventional lipid parameters are essential for diagnosing FH, they do not fully capture the metabolic impact of inherited *LDLR* dysfunction. This study aimed to characterize the plasma lipidomic phenotype associated with pathogenic *LDLR* variants in individuals with suspected FH. Forty-four participants underwent genetic testing for *LDLR,* apolipoprotein B-100 (*APOB*), and protein convertase subtilisin/kexin type 9 (*PCSK9*). After excluding three carriers of the *APOB* p.Arg3527Gln variant, the comparative lipidomic analysis included 41 participants. The study included 17 carriers of pathogenic *LDLR* variants and 24 participants without a detected pathogenic variant in the tested panel. We performed plasma lipidomic profiling using flow-injection analysis coupled with high-resolution tandem mass spectrometry (FIA-MS/MSALL) without chromatographic separation. Lipid annotation and data processing were performed using LipidView software, and lipidomic measurements were expressed as relative signal abundances derived from mass spectrometry (MS) peak intensities. Statistical analyses included Mann–Whitney U tests with false discovery rate (FDR) correction across the analyzed lipid features. Exploratory multivariable logistic regression and receiver operating characteristic (ROC) analyses were also performed. There were no differences in conventional lipid parameters between genetically confirmed *LDLR* variant carriers and participants without a detected pathogenic variant in the tested panel. At the nominal level (unadjusted *p* < 0.05), lipidomic profiling revealed differences in glycerophospholipid (GP) related analytical features between groups. However, no individual lipid feature remained statistically significant after full-family FDR correction. Among (GPs), 69 feature records (16.3%) showed nominal between-group differences, predominantly lower abundance in *LDLR* variant carriers. The most consistent nominal alterations were observed in features annotated as phosphatidylinositol triphosphates (PIP3) and N-acyl phosphatidylethanolamines (NAPE). Among the analyzed fatty acyls (FAs), 72.7% showed nominally lower relative abundance in *LDLR* variant carriers. We used exploratory multivariable models to identify GP and FA-related features associated with the detected pathogenic *LDLR* variant status. The models showed within-cohort discrimination. However, these models were developed and evaluated in the same small cohort after data-driven variable selection. They were not externally validated. Therefore, the findings should be interpreted as hypothesis-generating and do not establish diagnostic performance, clinical utility, or functional pathway impairment. Further validation in larger cohorts using targeted lipidomics with chromatographic separation is necessary.

## 1. Introduction

Lipid homeostasis is fundamental to cardiovascular health. It regulates processes such as lipoprotein metabolism, membrane integrity, intracellular signaling, inflammation, and vascular function [1]. In addition to their structural and energetic functions, lipids act as bioactive molecules impacting endothelial function, oxidative stress, immune responses, and vascular remodeling [2]. These processes contribute to the development and progression of atherosclerotic cardiovascular disease (ASCVD) [3]. FH, one of the most common inherited metabolic disorders, affects approximately one in 250 individuals and substantially increases their lifetime risk of premature ASCVD [4,5]. FH is most frequently caused by pathogenic variants in the *LDLR*, *APOB*, or *PCSK9* genes. These variants result in impaired LDL receptor-mediated clearance of circulating low-density lipoproteins (LDL-C), leading to lifelong exposure to elevated LDL cholesterol (LDL-C) concentrations [6,7,8,9]. Persistent LDL-C accumulation promotes endothelial dysfunction, chronic vascular inflammation, and accelerated atherogenesis. However, despite considerable advances in molecular diagnostics, a substantial proportion of patients who meet the clinical criteria for FH remain undiagnosed after standard genetic testing [9,10]. This suggests that additional metabolic mechanisms contribute to variability in disease expression among individuals. Recent large-scale clinical analyses continue to emphasize the substantial genetic and phenotypic heterogeneity of FH, highlighting the need for molecular approaches that complement conventional lipid measurements [11,12]. Traditional lipid parameters, such as total cholesterol, LDL-C, HDL-C, and triglycerides (TAGs), are still important for diagnosing and managing FH. However, they only partially reflect the biological processes underlying lipid metabolism and CVR [13,14]. In recent years, lipidomics has emerged as a powerful analytical approach that enables the simultaneous characterization of hundreds of lipid species [13]. High-resolution mass spectrometry has revealed that cardiovascular disease is associated with the coordinated remodeling of multiple lipid classes, extending beyond changes detectable by routine biochemical measurements. In a large, population-based study, Rämö et al. identified over one hundred lipid species associated with LDL-C and triglyceride concentrations. This finding underscores the intricacy of lipid remodeling beyond conventional lipid parameters and its correlation with coronary artery disease risk [15]. Similarly, De Simone et al. demonstrated that genetically confirmed heterozygous FH is associated with a distinct plasma lipidomic profile, particularly in phospholipid and sphingomyelin metabolism. These findings suggest that molecular lipid profiling could improve characterization of CVR beyond traditional lipid biomarkers [16,17,18,19]. In a previous lipidomic study, we demonstrated that individuals at very high cardiovascular risk (VHCVR), including patients with clinically suspected FH without detectable pathogenic variants in *LDLR*, *APOB*, or *PCSK9*, exhibited early alterations predominantly affecting GP metabolism despite only modest differences in conventional lipid parameters. These results imply that molecular lipid remodeling may precede abnormalities detectable by routine lipid testing and represent an early indicator of CVR [20]. Despite recent advances, the lipidomic consequences of pathogenic *LDLR* variants are not fully understood. In particular, information regarding the coordinated alterations of negatively charged (GPs), such as PIP3s and NAPEs, as well as oxygenated fatty acid species, is limited. These compounds play essential roles in membrane organization, intracellular signaling, mitochondrial function, and inflammatory regulation [21,22,23,24,25]. Therefore, we hypothesized that pathogenic *LDLR* variants are associated with the coordinated remodeling of interconnected lipid pathways that extend beyond conventional cholesterol metabolism. Accordingly, the present study combined next-generation sequencing (NGS) with an untargeted FIA-MS/MSALL workflow without chromatographic separation for lipidomics to characterize the plasma lipidomic phenotype associated with pathogenic *LDLR* variants in patients with clinically suspected FH. Furthermore, we sought to determine whether comprehensive lipidomic profiling captures genotype-associated metabolic alterations not reflected by conventional lipid parameters. This would contribute to a more comprehensive molecular characterization of *LDLR*-associated FH. The primary aim of this study was to characterize the plasma lipidomic phenotype associated with pathogenic *LDLR* variants in patients with clinically suspected FH and to explore whether lipidomic profiling reveals genotype-associated metabolic variation not captured by conventional lipid measurements.

## 2. Results

### 2.1. Genetic Testing, Study Population, and Clinical Characteristics

**Figure 1:**
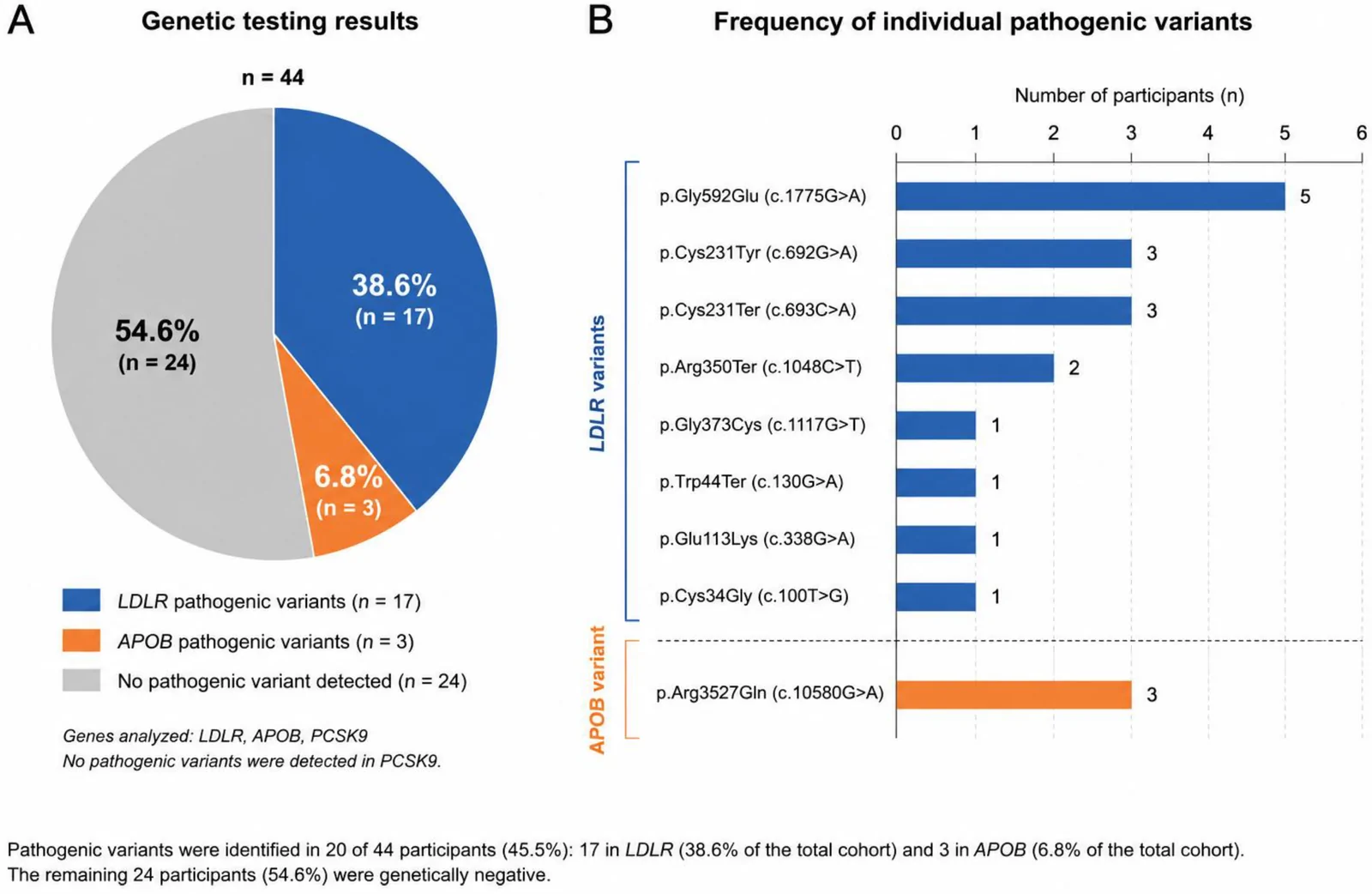
Distribution of pathogenic variants identified in the study cohort: (**A**) Distribution of pathogenic variants according to FH-associated genes. Twenty of the 44 participants (45.4%) were identified as carriers of pathogenic variants, including 17 carriers of *LDLR* variants and three carriers of an *APOB* variant. No pathogenic *PCSK9* variants were detected. (**B**) Distribution of individual pathogenic variants identified among genetically confirmed participants.

A pathogenic variant was identified in 20 patients (46%). Of those patients, 17 (85%) carried *LDLR* variants, and three (15%) carried an *APOB* variant (see Figure 1A). No pathogenic variants were identified in the *PCSK9* gene. Overall, nine distinct pathogenic variants were detected, including eight *LDLR* variants and one *APOB* variant (Figure 1B). The 17 *LDLR* variant carriers represented eight distinct variants and were analyzed as one group. No variant-specific lipidomic comparisons were performed. All of the identified variants had been previously reported in patients with FH. 

#### Participants with Versus Without a Detected Pathogenic Variant in the Tested Panel

Preliminary analyses revealed that *APOB* variant carriers (*n* = 3) formed a distinct subgroup with characteristics different from those of *LDLR* variant carriers. However, due to the small number of *APOB* variant carriers, this subgroup lacked sufficient statistical power for independent analyses and was excluded from subsequent analyses.

### 2.2. Lipidomic Profiling

#### 2.2.1. Differences in Lipid Profile in Female Subgroup

**Figure 2:**
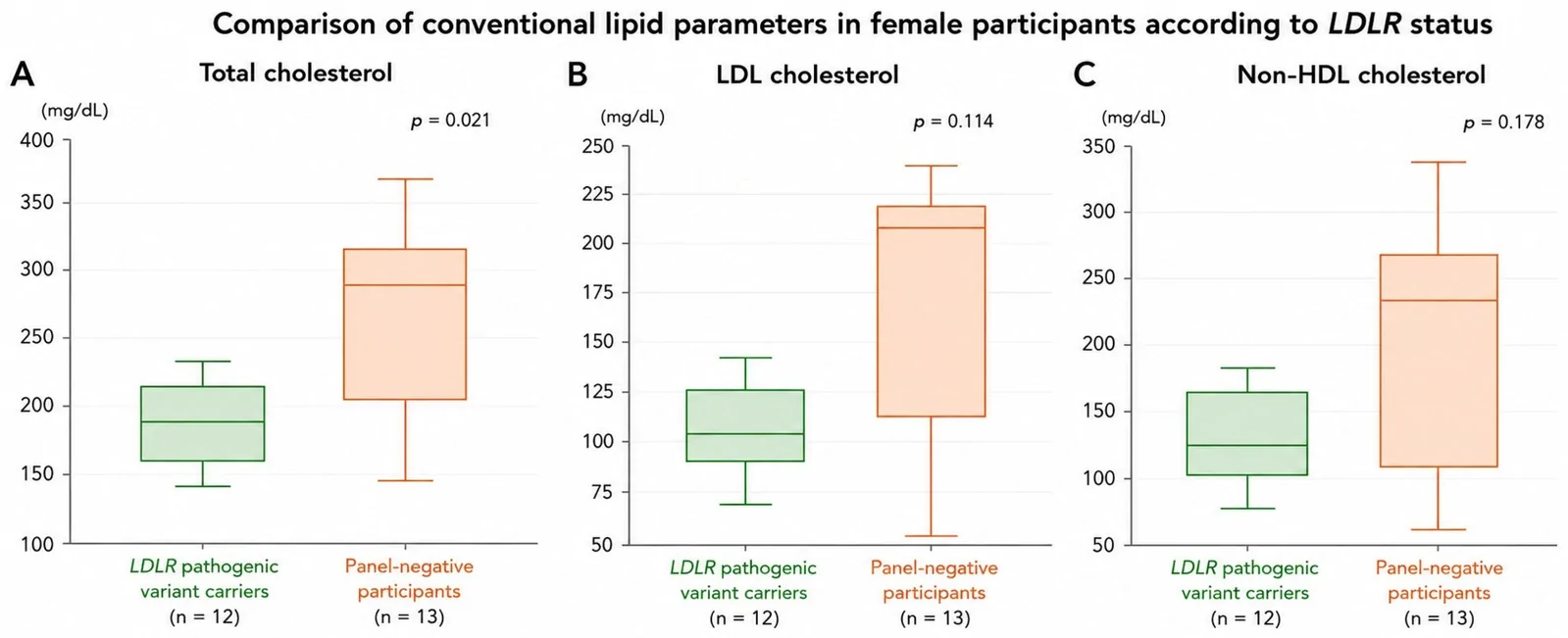
Conventional lipid profile by *LDLR* status in female participants. Boxplots of conventional lipid parameters in female participants by *LDLR* status: (**A**) Total Cholesterol, (**B**) LDL-C, and (**C**) Non-HDL cholesterol in women with pathogenic *LDLR* variants (*n* = 12) and those with no variants (*n* = 13). The horizontal lines show the median, the boxes show the interquartile range (IQR), and the whiskers indicate the minimum and maximum values within 1.5× IQR. *p*-Values were calculated using the Mann–Whitney U test. A two-way analysis (ROBUSTREG) performed on the entire patient group revealed statistically significant associations between cholesterol levels and sex (*p* < 0.001), and between cholesterol levels and the presence of an *LDLR* pathogenic variant (*p* = 0.003). No interaction between sex and the pathogenic variant was observed (*p* = 0.154).

In the post hoc subgroup analysis of women, total cholesterol levels were higher in participants without detected pathogenic variants than in *LDLR* variant carriers (median: 293 versus 193 mg/dL; nominal *p* = 0.021; see Figure 2A). No nominal differences were observed for LDL-C (*p* = 0.114; see Figure 2B) or non-HDL cholesterol (*p* = 0.178; see Figure 2C). However, the sex by *LDLR* variant interaction was not statistically significant (*p* = 0.154). Therefore, these findings should not be interpreted as evidence of a sex-specific association.

#### 2.2.2. Plasma Lipidomic Alterations Associated with Detected *LDLR* Variants

Plasma lipidomics revealed exploratory, nominal differences in the relative abundance of lipid signals between participants with and without detected pathogenic *LDLR* variants. The study analyzed over 700 lipid species, including various classes such as GP, sphingolipids (SP), and FA. The results showed minor differences in plasma lipid composition among carriers depending on the lipid class. GP exhibited the greatest number of altered lipid species, while FA had the highest proportion of altered compounds. SP, glycerolipids (GLs), and sterols (STs) showed few nominally altered species. Table 1 summarizes the nominally altered lipid species found in these classes.

#### 2.2.3. Glycerophospholipid Alterations Associated with Pathogenic *LDLR* Variants

GPs were the lipid class with the greatest number of nominally altered features in the negative-ion dataset. Of the 423 GP feature records included in the initial analysis, 69 (16.3%) showed nominal differences between participants with pathogenic *LDLR* variants and those with negative panels. Of these features, 72.5% exhibited lower relative MS signal abundance in *LDLR* variant carriers, and 27.5% exhibited higher relative MS signal abundance. FC ranged from 0.01 to 0.82 for features with lower signal abundance and from 1.18 to 1.97 for features with higher signal abundance.

The largest number of nominal findings was observed among features annotated as PIP3 and NAPE. Specifically, 14 out of 18 analytical feature records (77.8%) annotated as PIP3 and nine out of 16 analytical feature records (56.2%) annotated as NAPE showed nominal between-group differences. However, because several PIP3-annotated records shared the same target *m*/*z*, they should not be interpreted as 14 independently identified PIP3 molecular species. All nominally altered features in these two annotation groups exhibited lower relative MS signal abundance in *LDLR* variant carriers. Since the analysis was performed using FIA-MS/MSALL without chromatographic separation, these assignments should be interpreted as lipid annotations supported by the acquired MS/MS data, rather than as definitive identifications of individual, positional, regioisomeric, or otherwise independently resolved molecular species.

Correction for multiple comparisons across all tests revealed no statistically significant associations. The minimum *p*-Value was 0.181 (see Table S2).

**Figure 3:**
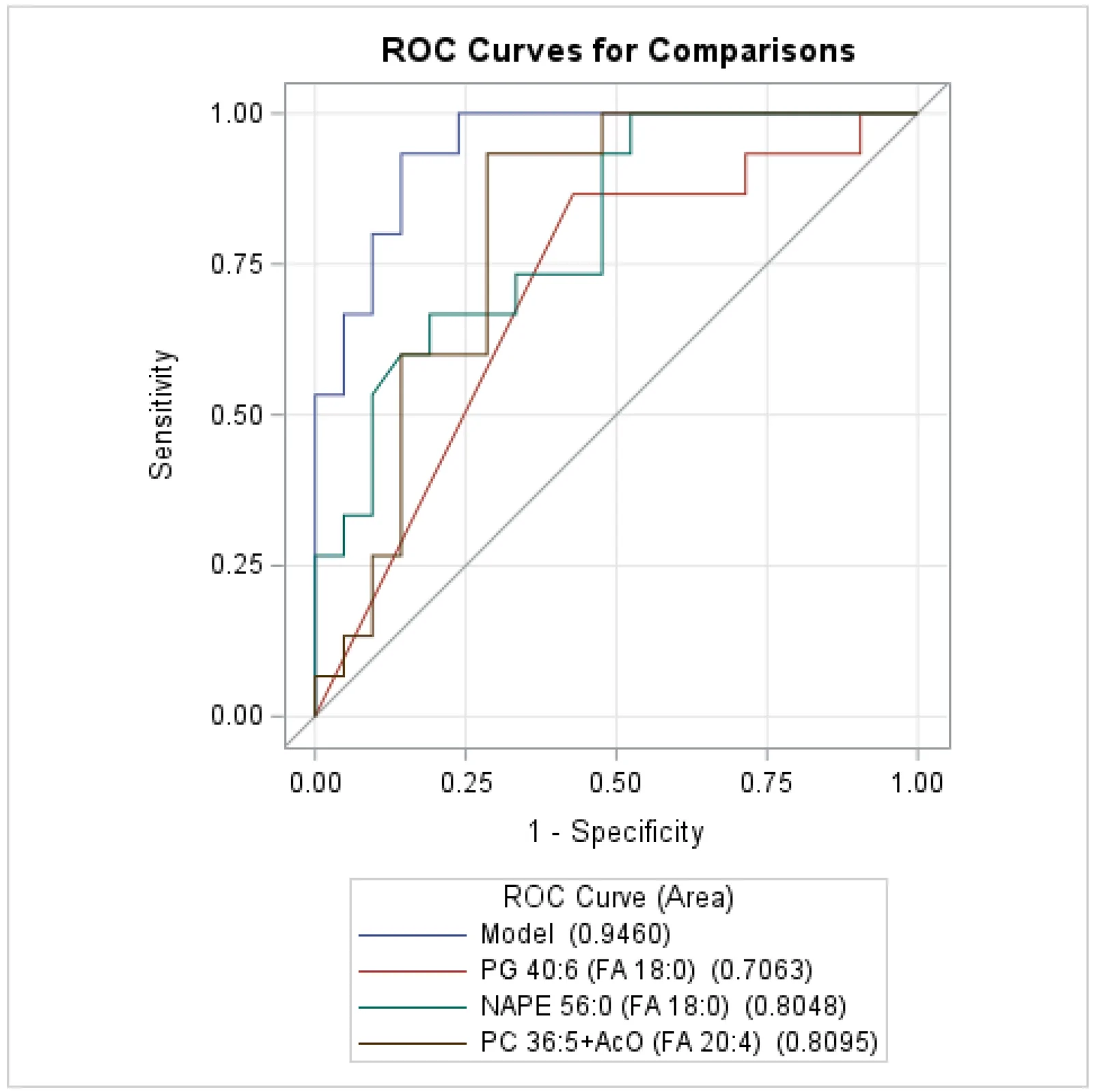
ROC curve analysis of exploratory lipid predictors of pathogenic *LDLR* variant status. PG—phosphatidylglycerol; NAPE—N-acyl phosphatidylethanolamines; PC—phosphatidylcholine.

Exploratory multivariable logistic regression identified three candidate predictors of *LDLR* variant status: phosphatidylglycerol (PG) (40:6/18:0), NAPE (56:0/18:0), and phosphatidylcholine (PC) (36:5/20:4). As described in Section 4.6, all three variables were transformed using log(x+1). No participants were excluded from the model due to zero values. The resulting exploratory model showed a within—cohort area under the curve (AUC) of 0.946 (95% confidence interval (CI): 0.880–0.999). However, the procedure using the SBC selection criterion identified PC (O-36:5/20:5) in addition to PG (40:6/18:0). Meanwhile, the models using ASE-based selection showed the significance of PG (40:8/18:2). The within-cohort AUC and apparent AUC (with 95% CL) for these models were 0.803 (95% CI: 0.661–0.946) and 0.841 (0.714–0.969), respectively. These results should be considered preliminary, though because the model was developed and evaluated in the same relatively small cohort and has not been externally validated. Figure 3 shows the discriminatory performance of the individual candidate features. Independent-cohort validation is required before these features can be considered candidate biomarkers.

In the positive-ion dataset, 11 out of 142 GP feature records (7.7%) showed nominal between-group differences. Seven of these features (63.6%) exhibited higher relative MS signal abundance in *LDLR* variant carriers, with fold changes ranging from 1.15 to 3.38. Conversely, four features (36.4%) exhibited lower relative MS signal abundance, with FCs ranging from 0.62 to 0.86. Correction for multiple comparisons across all tests revealed no statistically significant associations (see Table S3).

Exploratory multivariable logistic regression identified one candidate predictor of *LDLR* variant status: PC (34:2) (Table 2).

#### 2.2.4. Fatty Acyl Alterations Linked to Pathogenic *LDLR* Variants

FAs exhibited the highest proportion of lipid features, with nominal differences observed between groups. At the nominal threshold of *p* < 0.05, eight of the 11 analyzed FA features (72.7%) exhibited differences between carriers and non-carriers of pathogenic *LDLR* variants. The relative abundance of these eight features was lower in carriers, with FCs ranging from 0.32 to 0.66. The median relative abundance of all 11 analyzed FA features was lower in carriers, with a mean FC of 0.55 and a range of 0.32–0.85. This consistent direction of change indicates a generally lower abundance of this lipid class among carriers of pathogenic *LDLR* variants. One of the eight species that differed nominally was the hydroxy fatty acid O-16:0 (OAHFA) which showed lower abundance in carriers in the univariate analysis (FC = 0.32; nominal *p* = 0.029). However, after correcting for multiple comparisons, none of the FA associations remained statistically significant (Table S4).

An exploratory LASSO-type multivariable logistic regression analysis was performed on log-transformed FAs using AIC, SBC, and ASE criteria. Analysis using the AIC identified two significant components: hydroxy fatty acid O-16:0 (OAHFA; *p* = 0.018) and OAHFA-18:1 (*p* = 0.033). These two components were associated with pathogenic *LDLR* variant status. The AIC model yielded a within—cohort area under the ROC curve of 0.813 (95% CI: 0.674–0.952). In contrast, the procedure using the SBC selection criterion identified OAHFA (18:1/20:2) as the only significant predictor, achieving a within—cohort AUC of 0.735 (95% CI: 0.570–0.899). Using the ASE selection criterion, the procedure identified OAHFA (16:0/18:1) as the only significant predictor and achieved an apparent AUC of 0.945 (95% CI: 0.855–0.999).

#### 2.2.5. Sphingolipid Alterations Linked to Pathogenic *LDLR* Variants

SP exhibited fewer changes associated with pathogenic *LDLR* variants than GP and FA. Twenty SP species were detected in negative ionization mode. Of these, 10.0% (two) showed nominal differences between carriers and non-carriers of pathogenic *LDLR* variants (unadjusted *p* < 0.05). Higher levels of these lipids were observed in carriers. Additional changes were seen in SP species in positive ionization mode, with five out of 54 (9.3%) showing nominal differences between the two groups. Four of these species (80%) exhibited higher abundance in carriers, with ratios ranging from 1.18 to 6.36, and one species (20%) exhibited lower abundance, with a ratio of 0.78. All of the nominally different positively ionized SP species were from the sphingomyelin (SM) subclass, accounting for 10.6% (five out of 47) of the SM species. In contrast, no nominal differences were observed among seven long-chain base (LCB) species. Correction for multiple comparisons across all tests revealed no statistically significant associations (see Tables S5 and S6).

While some SP species showed nominal differences in univariate analysis, they did not predict pathogenic low-density lipoprotein receptor (*LDLR*) variant status in multivariate analysis.

#### 2.2.6. Alterations to Glycerolipids Linked to Pathogenic *LDLR* Variants

Compared to other lipid classes, GL exhibited minimal changes associated with pathogenic *LDLR* variants. Of the 58 GL species studied, only one (1.7%) showed nominal differences between individuals with and without these variants. TAG (54:6) was present at a lower relative abundance in variant carriers, with a fold change of 0.40. This nominal difference was found exclusively within the TAG subclass of one of the 32 TAG species (3.1%). Correction for multiple comparisons across all tests revealed no statistically significant associations (Table S6).

#### 2.2.7. Sterol Alterations Associated with Pathogenic *LDLR* Variants

Of the 14 analyzed sterol species, only one compound, cholesteryl ester (CE) (20:4), showed a nominal difference between individuals with and without pathogenic *LDLR* variants. CE (20:4) was higher in *LDLR* variant carriers, with an FC of 1.35. No other ST species showed nominal differences between the study groups. Correction for multiple comparisons across all tests revealed no statistically significant associations (see Table S6).

#### 2.2.8. Sensitivity Analysis After Excluding Related Individuals

To evaluate the robustness of the results, the final models were re-evaluated after excluding two participants who enrolled in the study later and were related to each other.

The sensitivity analysis regarding PG confirmed the statistical significance of PG (40:6/18:0), NAPE (56:0/18:0), and PC (36:5/20:4). These features were identified using an Akaike’s information criterion (AIC)-based model selection approach (*p* = 0.021, *p* = 0.036 and *p* = 0.031, respectively). The model achieved an AUC = 0.949 within the cohort. Two additional models showed the significance of PG (40:6/18:0), Schwarz’s Bayesian criterion (SBC, *p* = 0.043) and PG (40:8/18:2), average squared error (ASE—based selection, *p* = 0.033); (Table 3).

Even after excluding two patients from the same families who were identified as subsequent cases, FAs exhibited the highest proportion of lipid features, with only nominal differences between the two groups (see Table S5). At the nominal threshold of *p* < 0.05, six of the 11 analyzed FA features (54.5%) differed between carriers and participants without a pathogenic *LDLR* variant. All six of these features exhibited lower relative abundances in carriers, with FCs ranging from 0.32 to 0.66. (Table S4a). This pattern suggests that the relative abundance of the selected FA features is lower in carriers of a pathogenic *LDLR* variant. Exploratory one-sided post hoc analyses were performed to further assess this hypothesis. The analysis revealed lower concentrations of eight OAHFA features (72.7%) in the pathogenic *LDLR* variant carrier group compared to the group in which the variant was not detected. Although none of the FAs were statistically significant after correction for multiple comparisons in a two-sided test, the one-tailed analysis revealed a similar pattern after correction.

A sensitivity analysis supported the associations of OAHFA (16:0/18:1) and OAHFA (18:1/18:1). These features were identified using an AIC-based model selection approach (*p* = 0.014 and *p* = 0.024, respectively). The model achieved an apparent within-cohort AUC of 0.809. However, the LASSO and SBC-type selection procedures did not reveal any significant associations. Using the LASSO and ASE-based selection procedure, OAHFA (18:1/20:2) was associated with pathogenic LDLR variant status (*p* = 0.020), with an apparent AUC of 0.887.

Supplementary:

See the Supplementary Materials for complete lists of all tested lipid features. The tables include relative abundance measures, fold changes, raw *p*-Values, full family (FDR)-adjusted *p*-Values, median abundances, and lipid annotations according to the Lipid and Marker Views and the LIPID MAPS nomenclature [26] (Tables S1–S6, and Figure S1).

## 3. Discussion

Traditionally, FH, a monogenic disorder stemming from a faulty LDL receptor, has relied on conventional lipid measurements and molecular testing for diagnosis and risk assessment. However, this study and other emerging evidence suggest that the plasma lipidomic phenotype associated with pathogenic *LDLR* variants may extend beyond conventional cholesterol measurements [10,11,13,16,18,27,28]. This exploratory, cross-sectional analysis identified nominal differences in relative plasma lipid signal abundances between groups according to pathogenic *LDLR* variant status. These findings do not demonstrate that *LDLR* dysfunction caused the observed lipid differences or establish a systemic biological mechanism. Our high-resolution, untargeted lipidomics analysis revealed an exploratory plasma lipidomic pattern associated with a pathogenic *LDLR* variant. This phenotype was characterized by differences concentrated in GPs and lower abundance of FA features among carriers. Interestingly, conventional lipid parameters were similar between *LDLR* mutation carriers and individuals with suspected FH lacking detected pathogenic variants. These results suggest that lipidomic profiling may provide additional information about metabolic variation not fully reflected by routine lipid measurements.

Our findings corroborate those of previous studies by Cerda et al. and Du et al., which demonstrated the usefulness of lipidomic profiling in identifying metabolic phenotypes in FH patients, predicting therapeutic responses, and highlighting the role of phospholipid metabolism in cholesterol homeostasis and vascular injury [29,30]. Our study builds on these observations by showing that multiple phospholipid subclass remodeling types are associated with pathogenic *LDLR* variants, even in patients with severe clinical phenotypes. A significant strength of our study is its comparative design. Unlike many previous lipidomic studies that contrasted FH patients with healthy controls, we compared two clinically similar groups with severe hypercholesterolemia that differed primarily in the presence of pathogenic *LDLR* variants. This approach reduces major clinical heterogeneity, enabling a more focused evaluation of lipidomic patterns associated with pathogenic *LDLR* variant status. However, residual confounding cannot be fully excluded.

Building on our previous study [20], which associated disturbances in GP metabolism with an increased risk of cardiovascular disease in individuals without pathogenic *LDLR*, *APOB*, or *PCSK9* variants, the present exploratory analysis suggests that phospholipid remodeling may be more pronounced in individuals with pathogenic *LDLR* variants. This pattern occurred alongside differences in signaling phospholipids and FA metabolism. Thus, while our previous work identified GP remodeling in relation to cardiovascular risk, the present study raises the hypothesis that inherited *LDLR* dysfunction contributes to this phenomenon. Together, these studies suggest that GP remodeling is a general molecular characteristic of cardiovascular disease that varies according to pathogenic *LDLR* variant status. It is important to note that participants without pathogenic variants should not be considered healthy controls. They met the same clinical criteria for suspected FH, but no pathogenic variant was detected in the tested genes. This study compares lipidomic differences associated with detected *LDLR* variant status within a clinically selected population suspected of having FH. It does not evaluate differences between FH patients and healthy individuals. Therefore, pathway-level interpretations must be regarded as hypotheses requiring longitudinal and experimental confirmation.

Our results suggest that pathogenic *LDLR* variant status is associated with variation across interconnected lipid pathways rather than with a single disease-specific biomarker. These findings support a systems biology approach to interpreting the metabolic consequences of inherited LDL receptor dysfunction [5,6,31]. This approach considers alterations in membrane architecture, intracellular signaling, mitochondrial lipid metabolism, and FA homeostasis simultaneously. This interpretation is consistent with the evolution of lipidomics, which shifts the focus from discovering biomarkers to characterizing disease-specific molecular phenotypes. It provides a conceptual framework for investigating the metabolic phenotype of FH beyond conventional cholesterol measurements. GP accounted for the largest number of different molecular species, and the GP exploratory model yielded the highest AUC among the evaluated models. These nominal differences involved multiple metabolically connected subclasses, suggesting a possible relationship between the status of pathogenic *LDLR* variants, membrane lipid composition, and lipid-mediated signaling pathways. GPs influence numerous processes associated with cardiovascular disease [13,14]. Evidence suggests that lipid remodeling disruptions contribute to endothelial dysfunction, chronic inflammation, oxidative stress, and atherosclerosis in FH patients.

The lower relative abundance of analytical features annotated as PIP3 is consistent with variations in phosphatidylinositol 3-kinase (PI3K)/Akt-related lipid signaling. However, this interpretation is hypothetical because pathway activity was not measured, and the analytical workflow does not permit definitive identification of individual PIP3 molecular species. Previous studies have shown that compromised PI3K/PIP3 signaling can contribute to endothelial dysfunction and atherosclerosis, even when circulating cholesterol levels are normal [21,24].

The lower abundance of PIP3-annotated features identified in this study justifies further investigation of this pathway. However, this does not demonstrate impairment of functional signaling. A similar pattern was observed for NAPE—annotated features, more than half of which showed lower relative abundance in *LDLR* variant carriers. NAPEs are precursors to N-acylethanolamines, which regulate inflammation, stress, and metabolism [22,23,25]. While NAPE metabolism is well understood in conditions such as obesity, diabetes, and metabolic syndrome, its role in FH is unclear.

Our findings suggest that NAPE metabolism is a relevant pathway for further study. However, they do not demonstrate impaired NAPE metabolism or a causal link with inflammation. The precise role of NAPE metabolism in disease progression or membrane remodeling requires further investigation. Including phosphatidylglycerol (PG) as a candidate variable in the exploratory model provides an additional perspective.

PG is a cardiolipin (CL) precursor and CL is the primary phospholipid found in the inner mitochondrial membrane. It is essential for respiration and oxidative phosphorylation [24]. CL remodeling has been associated with mitochondrial dysfunction, oxidative stress, heart damage, and cardiovascular disease progression. The observed PG differences may be compatible with variations in mitochondrial membrane lipid composition associated with *LDLR* variant status. However, since mitochondrial function and energy metabolism were not measured directly, these observations justify targeted mechanistic studies rather than providing evidence of mitochondrial dysfunction. An exploratory multivariable model incorporating PG (40:6/18:0), NAPE (56:0/18:0), and PC (36:5/20:4) produced an AUC value of 0.946 (95% confidence interval (CI): 0.880–0.999). This value reflects the apparent separation within the analyzed cohort. However, since the model was developed and evaluated in the same small cohort, and since variable selection was based on the data, the reported AUC represents apparent within-cohort discrimination rather than externally validated diagnostic performance. Therefore, it should be interpreted cautiously due to the risk of overfitting. Independent validation using a prespecified or penalized model, together with internal resampling or nested cross-validation, is essential before any clinical interpretation. While the model composition may generate hypotheses for future targeted studies, it does not confirm that these phospholipids are diagnostic biomarkers. The second major finding was that free fatty acids (FFAs) exhibited the highest proportion of different features, all of which were present in lower quantities in individuals with pathogenic *LDLR* variants [18,28].

This consistent pattern is compatible with a broader class-level trend. FAs are crucial substrates for membrane lipid synthesis, β-oxidation, and intracellular signaling. Alterations in FA metabolism have been associated with mitochondrial dysfunction, oxidative stress, and endothelial injury [32]. An exploratory multivariable analysis identified hydroxy fatty acid O-16:0 (OAHFA) and OAHFA-18:1 as potential predictors of *LDLR* variant status. The resulting model had an apparent AUC of 0.816 (95% confidence interval (CI): 0.678–0.954). While these findings generate hypotheses, they do not establish that the OAHFA features constitute a reproducible or clinically relevant component of the lipidomic phenotype associated with pathogenic *LDLR* variants. These results support further research into FA homeostasis in FH. However, these findings do not establish whether the observed differences reflect a specific adaptive or pathological mechanism. The results suggest a link between a particular genetic variation and lipid alterations that extend beyond the scope of conventional cholesterol assessments. This expands the understanding of FH beyond routine cholesterol readings, though it does not prove the presence of structural membrane alterations. Further investigation is needed for confirmation. While conventional methods are still important for diagnosis and treatment, these findings underscore their limitations in capturing all plasma lipidomic differences related to pathogenic *LDLR* variant status. Lipidomic profiling may offer additional insights, though its role in diagnosis or treatment remains undefined. These observations apply to multi-omic cardiovascular research, but further validation is required for clinical interpretation [31].

One strength of our study is that it contrasts patients with severe hypercholesterolemia according to their genetic status. This design allows for a more targeted evaluation of metabolic patterns associated with pathogenic *LDLR* variant status. Our study also builds on previous research indicating that GP remodeling may be a characteristic of cardiovascular disease and monogenic disorders of lipid metabolism. The phospholipid differences observed here should be regarded as a candidate metabolic pattern requiring confirmation. This exploratory study compared lipidomic profiles between two groups that differed in *LDLR* variant status. The cohort included 17 carriers of eight distinct pathogenic *LDLR* variants, reflecting FH’s genetic heterogeneity. However, meaningful comparisons between variant subgroups were not possible due to their small size. Additionally, some participants came from two families, so a certain amount of within-family similarity cannot be ruled out. Larger, independent cohorts would help refine estimates and assess generalizability. While a cross-sectional design enables the identification of associations, it does not establish temporal sequence or causality. Nevertheless, the findings provide evidence to generate hypotheses about a plasma lipidomic phenotype linked to pathogenic *LDLR* variants.

Additionally, while untargeted lipidomics provides broad characterization, it offers relative rather than absolute quantification. Future studies employing targeted lipidomics are essential for a more direct and precise assessment [33,34].

Preliminary analyses indicated that including *APOB* p.Arg3527Gln carriers reduced the nominal significance of several lipid species and the overall separation between study groups [9,10,11]. This finding supports evaluating *APOB* carriers separately. These results imply that distinct molecular lipidomic phenotypes may characterize different monogenic forms of FH. Further investigation is necessary to improve our understanding of FH heterogeneity and to identify genotype-associated metabolic phenotypes.

In summary, this exploratory, cross-sectional analysis identified nominal differences in relative plasma lipid signal abundances between groups according to pathogenic *LDLR* variant status. The findings suggest a candidate metabolic phenotype involving several lipid classes, particularly glycerophospholipid-related features. However, these results do not establish causality, pathway dysfunction, structural membrane changes, or diagnostic utility. These observations clearly support further evaluating lipidomics in cardiovascular research. However, they do not demonstrate clinical utility or diagnostic performance.

### Study Limitations

This single-center study has several limitations. First, the sample size is small, with only 17 participants carrying pathogenic *LDLR* variants and 24 panel-negative participants. Thus, its findings may not be generalizable. The study was designed to compare lipidomic patterns between two groups of patients with suspected FH who differed in their genetic status. Blood samples were collected at the start of the study. Although statin use was balanced between the groups (17.6% versus 20.8%), the influence of lipid-lowering therapy on plasma lipid composition cannot be entirely ruled out. Importantly, blood samples were obtained during the participants initial visit to the lipid clinic. Most individuals had not yet started lipid-lowering therapy when the samples were collected. The remaining participants received treatment at comparable frequencies in both study groups. Therefore, although most participants had not started lipid-lowering therapy at the time of blood collection, and treatment frequencies were similar between groups, treatment-related confounding factors cannot be excluded. The participants were a clinically heterogeneous group with respect to cardiovascular comorbidities. Information on hypertension, coronary artery disease, carotid atherosclerosis, chronic kidney disease, impaired glucose metabolism, type 2 diabetes mellitus, and thyroid disorders was available and is presented in the baseline characteristics. According to the available clinical records, all participants had a body mass index (BMI) below 25 kg/m^2^. However, standardized and complete data regarding several lifestyle and treatment-related factors, including smoking status, dietary habits, and the duration and intensity of lipid-lowering therapy, was unavailable for some participants. Consequently, these variables could not be incorporated into multivariable adjustment models. Although statin use was balanced between groups, residual confounding factors related to treatment and lifestyle cannot be fully excluded. Therefore, the observed lipidomic differences should be interpreted with caution and confirmed in larger, prospectively phenotyped cohorts. Four carriers of pathogenic *LDLR* variants from two families were included. However, some degree of similarity in lipid profiles within families cannot be ruled out because relatives may share genetic and lifestyle characteristics, reducing the independence of individual observations. Including these four participants reflects the recruitment setting and enables the assessment of patients with rare pathogenic variants. A larger cohort would strengthen the evidence for confirmation.

A sensitivity analysis excluding two related participants, yielded similar results. Nevertheless, larger, independent cohorts are needed to confirm these findings.

The 17 carriers represented eight different pathogenic *LDLR* variants, creating small, unequal subgroups. Comparisons between variants are not meaningful. Therefore, the present findings represent an aggregate lipidomic phenotype associated with the presence of the detected pathogenic *LDLR* variants rather than the variants themselves. Larger, multicenter cohorts are needed to determine if different *LDLR* variants are associated with lipidomic patterns.

The definitions of comparator groups are important. Participants without a detected pathogenic variant met the same clinical criteria for suspected FH, and no pathogenic variants were detected in their *LDLR*, *APOB*, or *PCSK9* coding and splice-region sequences [9,10,35]. All participants met the clinical criteria for suspected familial hypercholesterolemia according to the Dutch Lipid Clinic Network (DLCN) score before genetic testing. Therefore, the comparator group should not be interpreted as a healthy control population but as a clinically matched group with an unresolved genetic background. This suggests the presence of other variants or genes that contribute to lipid metabolic processes. Thus, comparisons reflect the detected *LDLR* variants rather than distinguishing genetic from non-genetic hypercholesterolemia.

Participants without the detected pathogenic variant were clinically matched to *LDLR* variant carriers and met the criteria for suspected FH. They should not be interpreted as healthy controls. This study evaluates metabolic patterns associated with the presence of a detected *LDLR* variant within a population suspected of having FH.

The number of lipid species analyzed requires careful statistical interpretation, so the observed associations should be regarded as exploratory. However, the consistent direction of the observed nominal differences within certain classes provides a structure to guide targeted validation. Multivariable GP and FA models describe cohort separation. These models were developed and evaluated in the same small cohort using data-driven variable selection. Reported AUC values reflect within-cohort discrimination and may not reflect performance outside the cohort. Univariate class- and subclass-level observations remain valid. Internal resampling and external validation in larger, independent cohorts are necessary before reproducibility can be determined. The cross-sectional design precludes establishing a causal relationship between pathogenic *LDLR* variants and lipid differences. Therefore, interpretations involving phospholipid signaling, mitochondrial membrane lipid composition, and FA homeostasis are hypothesis-based and do not demonstrate impairment. Further studies and experimental investigations are needed to determine how these lipidomic patterns develop and whether they are related to LDL receptor dysfunction. Untargeted lipidomics quantifies relative amounts. Assays are needed to confirm selected species and ensure reproducibility [33,34].

This study did not evaluate the accuracy or performance of clinical biomarkers. ROC models do not replace genetic testing. Rather, they provide a metabolic characterization of FH. This serves as a foundation for future studies. Future studies in larger cohorts are planned and will incorporate chromatographic lipidomic workflows with prior chromatographic separation. These improvements will enhance the confidence and resolution of structural annotation and the resolution of isomeric and isobaric lipid species.

Lipid annotations were assigned using information from the precursor ion and MS/MS fragments, as well as by comparing them with the lipid fragment database in LipidView. However, since the FIA-MS/MSALL workflow was performed without chromatographic separation, unambiguous resolution of positional isomers, regioisomers, and certain isobaric lipid species is not possible. Therefore, the reported lipid assignments should be considered MS/MS-supported structural annotations permitted by the acquired data, rather than definitive identifications of specific positional or regioisomeric forms. This limitation is particularly relevant interpreting the PIP3, NAPE, and OAHFA-annotated features highlighted in the present study. Confirmation of these annotations and resolution of individual isomeric species would require targeted analyses using chromatographic separation and appropriate reference standards.

## 4. Materials and Methods

### 4.1. Study Population

A total of 44 consecutive adult patients with suspected FH were prospectively enrolled at the Outpatient Lipid Clinic of the National Institute of Cardiology in Warsaw. Blood samples were collected for genetic and lipidomic analyses during the initial study visit. Pathogenic variants were identified in 20 participants, including 17 carriers of the *LDLR* variant and three carriers of the *APOB* p.Arg3527Gln variant. However, the *APOB* subgroup was too small for meaningful comparative analysis, representing a distinct molecular mechanism. Thus, the three *APOB* carriers were excluded from the lipidomic comparison. The final analytical cohort therefore comprised 41 participants: 17 *LDLR* variant carriers and 24 participants without pathogenic variants in the tested genes. Nevertheless, the group without detected pathogenic variants should not be interpreted as a healthy control group. These participants exhibited clinical signs of FH, but no pathogenic variants were detected in the tested genes. Thus, the comparison evaluates differences in the lipidome associated with the presence or absence of a detected *LDLR* variant rather than differences between individuals with genetically defined FH and healthy individuals. Eight participants (19.5%) were on statin therapy, with an equal distribution between *LDLR* variant carriers and participants with no detected pathogenic variants (17.6% vs. 20.8%; *p*-Value = 1.000). According to the 2021 European Society of Cardiology (ESC) guidelines on cardiovascular disease prevention [36], all participants were classified as having high or very high cardiovascular risk. Despite routine clinical management, they presented with elevated total and LDL-C concentrations. Clinically suspected FH was defined according to the DLCN criteria. Individuals with a DLCN score indicating possible, probable, or definite FH were considered eligible for inclusion and subsequently underwent genetic testing. A final diagnosis of genetically confirmed FH was based on the identification of pathogenic or likely pathogenic variants in the *LDLR, APOB,* or *PCSK9* genes [37].

The inclusion criteria were: (1) age ≥18 years, (2) clinically suspected FH according to the DLCN based on lipid profile and clinical evaluation, and (3) high or very high cardiovascular risk. The exclusion criteria were: (1) lack of written informed consent and (2) age below 18 years.

The study population consisted of 25 women and 19 men with a mean age of 50.6 ± 10.3 years. Baseline demographic and clinical characteristics are presented in Table 4.

All participants underwent NGS of the *LDLR*, *APOB*, and *PCSK9* genes. Pathogenic variants were identified in 20 of the 44 enrolled participants (45.5%), including 17 carriers of *LDLR* variants and three carriers of the *APOB* p.Arg3527Gln variant. No pathogenic variants were detected in the *PCSK9* gene. Of the 17 *LDLR* variant carriers included in the lipidomic analyses, four were from two families. Two carried the *LDLR* c.692G>A (p.Cys231Tyr) variant and two carried the *LDLR* c.1048C>T (p.Arg350Ter) variant.

Since only three participants carried *APOB* variants, this subgroup was excluded from subsequent comparative lipidomic analyses. This decision was made to obtain a biologically homogeneous study population and to avoid potential bias arising from the distinct molecular mechanisms underlying *LDLR*- and *APOB*-associated FH. Additionally, the small number of *APOB* mutation carriers prevented meaningful statistical analysis of this subgroup.

According to the available clinical records, all participants had a BMI below 25 kg/m^2^ at the time of assessment.

### 4.2. Blood Sampling and DNA Extraction

Peripheral venous blood samples were collected from all participants after an overnight fast. The samples were collected in tubes containing ethylenediaminetetraacetic acid (EDTA). The samples were processed immediately after collection. Whole blood was used to isolate genomic DNA, and plasma was separated by centrifugation at 2000× *g* for 15 min at room temperature (5702R: Eppendorf, Hamburg, Germany). The plasma was then aliquoted and stored at −80 °C until lipidomic analysis (TSE320VGP: Thermo Scientific, Thermo Fisher Scientific, Bremen, Germany). Samples were analyzed after the study cohort was fully recruited; consequently, the maximum storage duration was approximately six months from the collection of the earliest samples. Repeated freeze–thaw cycles were avoided.

Genomic DNA was isolated from whole blood using the MagCore^®^ Genomic DNA Large Volume Whole Blood Kit (RBC Bioscience Corp., New Taipei City, Taiwan) according to the manufacturer’s instructions. Automated DNA extraction was performed using the MagCore^®^ Plus II Automated Nucleic Acid Extractor (RBC Bioscience Corp., New Taipei City, Taiwan). The purified genomic DNA was then used for NGS of the *LDLR*, *APOB*, and *PCSK9* genes.

### 4.3. Plasma Lipid Extraction

Venous blood samples were collected from overnight fasted subjects. Then, an aliquot of the 75 µL of experimental plasma samples was transferred to glass culture tubes (3 DRAM, 19 × 65 ST, Finneran, Vineland, NJ, USA). Then, 0.9 mL of water, 2 mL of methanol, and 0.9 mL of dichloromethane (Chempur, Piekary Śląskie, Poland) were added to each sample. The samples were gently vortexed—for five seconds using a (V-1 plus: Vortex, Biogenet, Warsaw, Poland) and then placed on the benchtop at room temperature (RT) for 30 min. Next, 1 mL of water and 0.9 mL of dichloromethane were added to the samples. The samples were gently vortexed for five seconds, after which they were centrifuged at 2800× *g* for 10 min at RT or until the extracts were visibly separated into a bilayer. Since over-vortexing at this stage could result in poor phase partitioning, the bottom of the organic layer was transferred to a new tube for each extract. Another 1.8 mL of dichloromethane was then added to the samples, which were gently vortexed for five seconds and centrifuged at 2800× *g* for ten minutes at RT or until the extracts were visibly separated into a bilayer. The combined bottom layers of each sample were concentrated under nitrogen and reconstituted in 0.25 mL of the running solution, which was 10 mM ammonium acetate (Merck, Darmstadt, Germany) and a (50/50 *v*/*v*) mixture of dichloromethane and methanol. The extracts were transferred to inserts and placed in vials (J0602: Chromsystems Instruments and Chemicals GmbH, Grafelfing, Germany) for FIA-MS/MSALL analysis. A modified protocol based on the Folch method of Lofgren et al. [39] was applied to the entire analysis.

### 4.4. Lipidomic Analysis

Using qualitative lipidomic analyses, more than 700 lipid compounds were analyzed and classified into different categories, including cholesterol esters, triglycerides, diglycerides, free fatty acids, bile acids, phosphatidylcholine, phosphatidylethanolamine, phosphatidylinositol, phosphatidylserine, sphingomyelins, and ceramides.

Lipidomic profiling was performed using a FIA-MS/MSALL workflow without chromatographic separation (Sciex, Framingham, MS, USA). Lipid extracts were introduced using an Exion LC AD system (Sciex, Framingham, MA, USA) operated in flow-injection mode. The autosampler was connected directly to the ion source via a 100-cm PEEKsil SecurityLINK capillary with a 50 μm internal diameter (Phenomenex, Torrance, CA, USA). The mobile phase was a (50:50 *v*/*v*) mixture of methylene chloride and methanol containing 5 mM ammonium acetate (LC/MS purity), with isocratic flow. To minimize carry-over between injections, the autosampler needle was washed with 100% isopropanol.

Mass spectrometric measurements were performed using an integrated analytical system consisting of a SCIEX TripleTOF^®^ 6600+ high-resolution mass spectrometer equipped with an OptiFlow^®^ ion source and a 65-μm electrode (Sciex, Framingham, MA, USA). Data were acquired in infusion MS/MSALL mode, comprising a TOF MS scan (5 s) followed by sequential MS/MS scans (300 ms). Fifty microliters of each sample extract were injected and analyzed over an *m*/*z* range of 400–1200. Data were acquired separately in positive and negative ionization modes using capillary voltages of +5000 V and −4500 V, respectively. Each sample was analyzed in technical triplicate for each polarity (six injections per sample total) to assess analytical repeatability. An exploratory principal component analysis (PCA)-based quality assessment was performed during data processing to evaluate the consistency of repeated measurements. The total analytical cycle, including the washing and conditioning steps, took approximately 12 min. Equilibration was performed from 0.0 to 1.2 min, data acquisition from 1.2 to 6.5 min (flow rate: 7 µL/min), and washing from 8.0 to 12.0 min (flow rate: 100 µL/min). The following source and gas parameters were used: a source temperature of 150 °C, a shielding gas of 25, a curtain gas 1 of 25, and a gas 2 of 30. The infusion MS/MSALL analysis yielded fragmentation spectra for all *m*/*z* values present in the sample. Raw MS/MS data were processed using LipidView™ software, version 1.2 (SCIEX). Lipid annotation was based on precursor-ion and MS/MS fragment information as well as a comparison with the lipid-fragment database in the software. The software provides information on expected molecular ions/adducts and characteristic fragment ions. It also incorporates isotope-correction factors during data processing. Statistical exploration and multivariate visualization were performed using MarkerView™ software, version 1.3 (SCIEX, Framingham, MA, USA). We performed an exploratory principal component analysis (PCA) in the MarkerView software to visualize global lipidomic variation and identify clustering trends. Since no isotope-labeled or lipid-class-specific internal standards were used, the lipidomic measurements represent relative signal abundances rather than absolute concentrations. Therefore, FCs describe relative differences in MS signal abundance between the two genetic groups. Observed values should not be interpreted as calibrated plasma concentrations. The FIA-MS/MSALL workflow does not provide chromatographic separation; therefore does not permit the unambiguous resolution of isomeric and certain isobaric lipid species. Consequently, lipid assignments should be interpreted as structural-level annotations supported by the acquired MS/MS data, rather than definitive identifications of individual positional or regioisomers [26]. This study is a secondary analysis of a lipidomic dataset previously generated and reported by Waś et al. [20]. However, the current analysis addresses a different biological question by stratifying participants according to *LDLR* genotype status.

### 4.5. Genetic Testing

Targeted NGS was performed on genes associated with FH. The analyzed regions included the promoter and all coding exons, as well as the adjacent intronic sequences of the *LDLR* gene; exon 26 of the *APOB* gene; and the coding exons and flanking splice-site regions of the *PCSK9* gene. Sequencing libraries were prepared using the Twist Library Preparation EF Kit (Twist Bioscience, South San Francisco, CA, USA). Target enrichment was performed using the Twist Hybridization and Wash Kit and a custom probe panel (Twist Bioscience, South San Francisco, CA, USA). Paired-end sequencing (2 × 100 bp) was carried out on a NovaSeq 6000 system (Illumina, Inc., San Diego, CA, USA). The mean depth of coverage was 1690×. Variants were classified according to the 2015 ACMG/AMP criteria [40], and ClinVar (National Library of Medicine, Bethesda, MD, USA) [41] was used as a supporting source for clinical interpretation. Only variants classified as pathogenic or likely pathogenic were used to define the molecularly positive groups.

### 4.6. Statistical Analysis

Due to the small sample size, comparisons of all numerical variables were made using the nonparametric Mann–Whitney test. The significance of proportions of categorical variables was verified using Fisher’s exact test. Since multiple tests increase the risk of a Type I error, correcting for multiple comparisons is advisable. However, the small sample size, non-normal lipid distributions (indicating the use of non-parametric methods), and large number of compounds compared within specific lipid groups reduce statistical power and the likelihood of obtaining a significant result after correction. This is true even when using a less conservative method than Bonferroni correction, such as FDR (PROC MULTTEST in statistical software (SAS), which controls the false discovery rate rather than the family-wise error rate. The supplementary tables provide complete statistical results for all analyzed lipid features, including fold changes, raw *p*-Values and full-family FDR—adjusted *p*-Values derived from the Mann–Whitney test. Subsequently, multivariable logistic regression was performed. To protect against overfitting, we used the LASSO method, which penalizes high regression coefficients. (PROC HPGENSELECT in SAS with the binomial distribution option). We applied three different selection methods: AIC, SBC, and ASE, computed from internal validation data (partition fraction: 0.3). For the ASE-based model selection process, we randomly assigned 25 observations to the training subset and 16 to the validation subset. The model with the lowest ASE in the validation set was selected as the optimal model. However, the ROC analyses presents an apparent AUC. First, we applied logistic regression with LASSO selection to the statistically significant variables identified in the preceding step (Mann–Whitney test results). Next, we constructed multivariable logistic regression models using stepwise selection to reduce multidimensionality and identify independent lipid predictors of pathogenic *LDLR* variant status. The initial model included the variables indicated by LASSO. Additionally, we calculated the odds ratios, their 95% confidence intervals, and *p*-Values for the significant predictors using Firth’s penalized likelihood method instead of classical maximum likelihood. Due to variability in lipid abundance measurements and the presence of zero values in some lipid features, we added a constant of 1 to all measurements prior to logarithmic transformation. Consequently, the variables included in the multivariable logistic regression models were transformed as log(x+1). This procedure allowed us to retain zero-valued observations in the analysis without excluding any participants due to undefined logarithmic values. Univariate ROC curves were constructed to evaluate the discriminatory performance of lipid species showing nominal differences between groups. The analyses presented for the group of women (Section 2.2.1) were performed post hoc rather than planned a priori. We analyzed the effects of gender and the *LDLR* pathogenic variant on total cholesterol levels using robust regression based on M-estimation (PROC ROBUSTREG in SAS). Interactions between gender and the *LDLR* pathogenic variant were also examined. The sensitivity analyses presented were conducted on the patient after excluding two cases that presented subsequently from the same family. All hypotheses were two-tailed, with a type I error rate of 0.05. One-sided post-hoc tests were also used in the sensitivity analysis. The statistical analyses were performed using SAS version 9.4.

## 5. Conclusions

This exploratory, cross-sectional study revealed minor differences in the relative abundances of plasma lipid signals based on the presence of identified pathogenic *LDLR* variants in a clinically selected population suspected of having FH. The findings suggest candidate patterns involving glycerophospholipid, PIP3, NAPE, and FA annotated features. However, these patterns do not establish causality, functional pathway impairment, diagnostic performance, or clinical utility. Exploratory models require appropriate internal validation and independent confirmation. Targeted lipidomics with chromatographic separation is necessary to confirm the structural identity and reproducibility of the candidate features.

## Figures and Tables

**Table 1:** Distribution of nominally altered lipid species in individuals carrying pathogenic *LDLR* variants compared to those without.

Ionization Mode/Lipid Class	Analyzed Lipid Species, *n*	Nominally Altered, *n* (%)	Higher in *LDLR* Variant Carriers, *n* (%)	Fold-Change Range	Lower in *LDLR* Variant Carriers, *n* (%)	Fold-Change Range
Negative ionization mode						
GP	423	69 (16.3)	19 (4.5)	1.18–1.97	50 (11.8)	0.01–0.82
SP	20	2 (10.0)	2 (10.0)	1.17–1.20	0 (0.0)	—
FA	11	8 (72.7)	0 (0.0)	—	8 (72.7)	0.32–0.66
Positive ionization mode						
GP	142	11 (7.7)	7 (4.9)	1.15–3.38	4 (2.8)	0.62–0.86
GL	58	1 (1.7)	0 (0.0)	—	1 (1.7)	0.40
SP	54	5 (9.3)	4 (7.4)	1.18–6.36	1 (1.9)	0.78
ST	14	1 (7.1)	1 (7.1)	1.35	0 (0.0)	—

The table summarizes the number and percentage of lipid species that were nominally altered in each class. It also includes the direction of the change and the range of the fold change (FC) range. Lipid species are grouped by ionization mode. Percentages are calculated relative to the total number of lipid species analyzed.

**Table 2:** The exploratory GP variables that were retained in the multivariable logistic regression model, which employed the LASSO method and three different selection procedures for pathogenic *LDLR* variant status, are presented below *. Odds ratios (ORs) and 95% confidence intervals (CIs) are reported for each 0.1-unit increase in the log(x + 1) transformed relative abundance of lipids. **—apparent AUC.

Variable	Odds Ratio (95% CI)	*p*-Value	Within-Cohort *AUC* (95% CL)
Model I (AIC)			
PG (40:6/18:0)	0.553 (0.334–0.914)	0.021	0.946 (0.880–0.999)
NAPE (56:0/18:0) *	0.687 (0.487–0.970)	0.033	
PC (36:5/20:4) *	1.765 (1.067–2.920)	0.027	
Model II (SBC)			
PG (40:6/18:0)	0.661 (0.440–0.906)	0.020	0.803 (0.661–0.946)
PC (O-36:5/20:5)	0.674 (0.446–0.946)	0.034	
Model III (ASE-based selection)			
PG (40:6/18:0)	0.660 (0.453–0.962)	0.030	0.841 (0.714–0.969) **
PG (40:8/18:2) *	0.755 (0.597–0.954)	0.019	

**Table 3:** Exploratory GP variables that were retained in the multivariable logistic regression model (using the LASSO method and three different selection procedures) for pathogenic *LDLR* variant status are presented below. * Odds ratios (ORs) and 95% confidence intervals (CIs) are reported for each 0.1-unit increase in the log(x + 1) -transformed relative abundance of lipids. **—apparent AUC.

Variables	Odds Ratio (95% CI)	*p*-Value	Within-Cohort *AUC* (95% CL)
Model I (AIC)			
PG (40:6/18:0)	0.456 (0.235–0.887)	0.021	0.949 (0.884–0.999)
NAPE (56:0/18:0) *	0.607 (0.381–0.968)	0.036	
PC (36:5/20:4) *	2.370 (1.084–5.180)	0.031	
Model II (SBC)			
PG (40:6/18:0)	0.717 (0.519–0.990)	0.043	0.784 (0.630–0.938)
PC (O-36:5/20:5)	0.736 (0.517–1.046)	0.088	
Model III (ASE—based selection)			
PG (40:6/18:0)	0.732 (0.521–1.030)	0.073	0.824 (0.683–0.965) **
PG (40:8/18:2) *	0.801 (0.653–0.982)	0.033	

**Table 4:** Baseline demographic, clinical, and conventional lipid profile characteristics of the study population according to *LDLR* pathogenic variant status data are presented as median (interquartile range (IQR)) or number (percentage) as appropriate. Continuous variables were compared using a Mann–Whitney U-test, depending on the data distribution. Categorical variables were compared using Fisher’s exact test. CA/CAD = atherosclerosis of the carotid or coronary arteries; CKD = chronic kidney disease; HDL-C = high-density lipoprotein cholesterol; IQR = interquartile range; LDL-C = low-density lipoprotein cholesterol; Non-HDL-C = non-high-density lipoprotein cholesterol; PAD = peripheral artery disease; T2DM = type 2 diabetes mellitus; TG = triglycerides; High cardiovascular risk was defined according to the POL-SCORE chart. Patients with diabetes mellitus and chronic kidney disease were considered to have a high cardiovascular risk. Patients with established cardiovascular disease were classified as having VHCVR [38].

Clinical Characteristics	All Participants (*n* = 41)	*LDLR* Pathogenic Variant Participants (*n* = 17)	No Detected Pathogenic Variant Participants (*n* = 24)	*p*-Value
Demographic Data				
Age (years), median (IQR)	51.7 (45–57)	49.3 (44–58)	52.5 (47–57)	0.552
Female sex, *n* (%)	25 (61.0)	12 (70.6)	13 (54.2)	0.288
CA/CAD, *n* (%)	3 (7.3)	2 (11.8)	1 (4.2)	0.560
PAD, *n* (%)	6 (14.6)	4 (23.5)	2 (8.3)	0.212
Hypertension, *n* (%)	10 (24.4)	5 (29.4)	5 (20.8)	0.714
CKD, *n* (%)	2 (4.9)	0 (0.0)	2 (8.3)	0.502
Impaired glucose metabolism, *n* (%)	5 (12.2)	1 (5.9)	4 (16.7)	0.382
T2DM, *n* (%)	1 (2.5)	1 (5.9)	0 (0.0)	0.415
Laboratory test results				
Total cholesterol (mg/dL), median (IQR)	202 (149–241)	168 (154–218)	214 (147–293)	0.341
LDL-C (mg/dL), median (IQR)	119 (70–192)	93 (74–130)	131 (63–217)	0.427
HDL-C (mg/dL), median (IQR)	53 (45–67)	59 (45–70)	53 (45–63)	0.549
Non-HDL-C (mg/dL), median (IQR)	131 (86–176)	118 (86–158)	144 (82–231)	0.496
TG (mg/dL), median (IQR)	133 (84–171)	128 (78–165)	137 (105–179)	0.390
Total cholesterol/HDL-C ratio, median (IQR)	3.0 (2.5–4.3)	3.0 (2.4–4.0)	3.1 (2.5–4.4)	0.481
LDL-C/HDL-C ratio, median (IQR)	1.6 (1.1–2.6)	1.6 (1.1–2.2)	1.6 (1.1–3.0)	0.540
Medication				
Statins, *n* (%)	8 (19.5)	3 (17.6)	5 (20.8)	1.000

## Data Availability

The data supporting the findings of this study are available from the corresponding author upon reasonable request. Summary data supporting the reported results are available from the authors. Patient data are unavailable due to privacy restrictions.

## Supplementary Materials

The following supporting information can be downloaded at: https://www.mdpi.com/article/10.3390/ijms27209011/s1.
