# Supplementary material for: Plasma Lipidomic Phenotype Associated with Pathogenic LDLR Variants in Familial Hypercholesterolemia
Source: Int J Mol Sci. 2026 Oct 11;27(20):9011. doi: 10.3390/ijms27209011 (PMC13705200; doi:10.3390/ijms27209011)
Supplement: Supplementary file 1 [file ijms-27-09011-s001.zip › Table S1.pdf]

Table S1. Lipid classes and abbreviations analyzed in this study.

Detailed results and fold-change values are presented in Tables S2–S6.

Glycerophospholipids (GP)	Sphingolipids (SP)	Fatty acyls (FA)
**PA** – Phosphatidic acid	**SM** – Sphingomyelin	**FFA** – Free fatty acid
**PC** – Phosphatidylcholine	**Cer** – Ceramide	**ME** – Methyl ester
**PE** – Phosphatidylethanolamine	**HexCer** – Hexosylceramide	**EE** – Ethyl ester
**PG** – Phosphatidylglycerol	**Hex2Cer** – Dihexosylceramide	**GlcdE** – Glycidol ester
**PI** – Phosphatidylinositol	**SHexCer** – Sulfatide	**OAHFA** – O-acyl-ω-hydroxy fatty acid
**PS** – Phosphatidylserine	**CerP** – Ceramide phosphate	
**LPC** – Lysophosphatidylcholine	**CerPE** – Ceramide phosphoethanolamine	
**CL** – Cardiolipin	**IPC** – Inositol phosphorylceramide	
**DGPP** – Diacylglycerol pyrophosphate	**MIPC** – Mannosyl-inositol phosphorylceramide	
**MMPE** – Monomethylphosphatidylethanolamine	**M(IP)2C** – Mannosyl-diinositol phosphorylceramide	
**DMPE** – Dimethylphosphatidylethanolamine	**Hex3Cer** – Trihexosylceramide	
**LDMPE** – Lysodimethylphosphatidylethanolamine	**GM3** – Monosialoganglioside	
**PIP** – Phosphatidylinositol phosphate	**GM2** – Monosialoganglioside-GalNAc	
**PIP2** – Phosphatidylinositol bisphosphate	**GM1** – Monosialoganglioside-Gal-GalNAc	
**PIP3** – Phosphatidylinositol trisphosphate	**GD3** – Disialoganglioside	
**CDP-DAG** – Cytidine diphosphate diacylglycerol	**GD2** – Disialoganglioside-GalNAc	
**NAPE** – N-acyl phosphatidylethanolamine	**GD1** – Disialoganglioside-Gal-GalNAc	
	**GT3** – Trisialoganglioside	
	**SGalCer** – Sulfogalactosylceramide	

Glycerolipids (GL)	Sterol lipids (ST)
**TAG** – Triacylglycerol	**CE** – Cholesteryl ester
**DGDG** – Digalactosyldiacylglycerol	

**MADAG** – Monoalkyl diacylglycerol	

Abbreviations: Gal, galactose; GalNAc, N-acetylgalactosamine.
