# Supplementary material for: Plasma Lipidomic Phenotype Associated with Pathogenic LDLR Variants in Familial Hypercholesterolemia
Source: Int J Mol Sci. 2026 Oct 11;27(20):9011. doi: 10.3390/ijms27209011 (PMC13705200; doi:10.3390/ijms27209011)
Supplement: Supplementary file 1 [file ijms-27-09011-s001.zip › Table S2.pdf]

Table S2. Comparison of glycerophospholipid relative abundances detected in negative ionization mode according to pathogenic *LDLR* variant status.

Lipid species	Carriers of pathogenic *LDLR* variant (n = 17)			Carriers with no detected pathogenic *LDLR* variant (n = 24)			Fold change	*p*-Value		Lipid annotation		
	Median	Q1	Q3	Median	Q1	Q3		Raw *p*-Value	FDR-adjusted *p*-Value	Scan m/z	m/z	Correction
				Cardiolipin (CL)								
CL (48:0/18:0)	130	88	234	235	158	351	0.56	0.029	0.237	283.275	563.413	1.514
CL (48:1/18:1)	121	78	238	288	166	417	0.42	0.037	0.249	281.260	562.412	1.978
CL (48:2/18:1)	468	269	1,008	1,406	495	1,677	0.33	0.027	0.223	281.260	561.435	1.978
CL (48:2/18:2)	510	307	1,075	1,470	498	1,804	0.35	0.047	0.286	279.244	561.435	1.978
CL (48:3/18:2)	41	26	55	87	50	136	0.47	0.025	0.218	279.241	560.374	1.977
CL (52:4/18:1)	62	32	105	106	67	171	0.58	0.027	0.223	281.258	587.437	2.068
CL (52:6/18:1)	133	58	302	297	132	458	0.45	0.037	0.249	281.259	585.422	2.067
CL (52:6/20:4)	85	38	103	120	60	149	0.71	0.047	0.286	303.243	585.422	2.067
CL (70:3/16:0)	130	114	175	198	159	238	0.65	0.007	0.181	255.243	714.592	2.533
CL (74:2/18:2)	417	365	553	575	492	690	0.72	0.006	0.181	279.243	743.636	2.650
CL (74:3/18:0)	290	234	333	377	338	515	0.77	0.012	0.181	283.274	742.572	2.650
CL (78:9/20:4)	725	608	930	598	422	725	1.21	0.045	0.284	303.244	764.598	2.769
CL (82:7/20:4)	479	398	642	386	338	465	1.24	0.018	0.181	303.244	794.592	2.798
CL (82:12/18:2)	249	206	325	333	265	412	0.75	0.012	0.181	279.243	789.586	2.798
CL (88:16/16:0)	215	176	255	167	131	191	1.29	0.018	0.181	255.242	827.677	2.809
CL (48:3/18:0)	221	113	358	234	149	357	0.95	0.531	0.532	283.274	560.374	1.977
CL (56:1/18:1)	14	0	61	57	32	106	0.24	0.162	0.162	281.259	618.504	2.165
CL (68:3/18:1)	342	247	444	330	288	383	1.03	0.847	0.847	281.259	695.545	2.474
CL (68:4/18:2)	111	79	189	142	105	181	0.78	0.352	0.352	279.243	699.549	2.476
CL (72:3/18:1)	459	315	563	402	350	481	1.14	0.422	0.422	281.259	728.585	2.591
CL (72:4/18:2)	199	136	303	244	169	276	0.82	0.500	0.500	279.243	730.599	2.592
CL (72:5/18:1)	312	261	352	267	216	319	1.17	0.127	0.127	281.259	726.576	2.550
CL (72:7/20:3)	223	163	297	229	176	256	0.98	1.000	1.000	305.259	724.559	2.589
CL (72:8/20:4)	560	403	829	592	426	687	0.95	0.797	0.797	303.244	723.549	2.588
CL (72:14/16:0)	109	71	159	150	113	225	0.73	0.109	0.109	255.242	717.612	2.585
CL (74:1/16:0)	233	202	249	243	216	277	0.96	0.386	0.386	255.242	733.583	2.645
CL (74:2/18:1)	299	182	393	312	250	432	0.96	0.386	0.386	281.258	743.636	2.650
CL (74:7/16:0)	344	272	368	415	299	468	0.83	0.109	0.109	255.242	738.588	2.648
CL (76:7/20:4)	433	330	583	348	321	472	1.25	0.378	0.378	303.244	752.587	2.708
CL (76:8/20:4)	1084	816	1647	944	812	1190	1.15	0.305	0.305	303.244	751.579	2.707
CL (76:9/22:4)	346	245	536	359	222	435	0.96	0.677	0.677	331.275	750.576	2.707
CL (76:10/20:4)	545	447	764	511	362	662	1.07	0.235	0.235	303.244	749.563	2.706
CL (76:11/22:5)	382	304	580	423	345	519	0.90	0.847	0.847	329.259	748.559	2.706
CL (76:13/18:0)	218	140	273	219	160	261	0.99	0.564	0.564	283.274	746.595	2.705

Lipid species	Carriers of pathogenic *LDLR* variant (n = 17)			Carriers with no detected pathogenic *LDLR* variant (n = 24)			Fold change	*p*-Value		Lipid annotation		
	Median	Q1	Q3	Median	Q1	Q3		Raw *p*-Value	FDR-adjusted *p*-Value	Scan m/z	m/z	Correction
CL (76:14/18:1)	307	278	349	346	286	392	0.89	0.235	0.235	281.259	745.583	2.705
CL (78:5/20:3)	378	334	443	453	361	526	0.83	0.153	0.153	305.259	768.582	2.771
CL (78:6/20:4)	805	686	913	823	710	1045	0.98	0.564	0.564	303.244	767.637	2.770
CL (78:7/18:0)	1146	1075	1420	1252	1037	1571	0.91	0.724	0.724	283.274	766.573	2.770
CL (78:7/20:4)	3092	2684	3693	3060	2803	4070	1.01	0.677	0.677	303.244	766.573	2.770
CL (78:9/18:1)	269	223	342	219	181	294	1.23	0.109	0.109	281.259	764.598	2.769
CL (78:9/20:5)	107	85	152	145	106	216	0.74	0.248	0.248	301.227	764.598	2.769
CL (78:11/16:0)	333	259	593	431	350	708	0.77	0.199	0.199	255.242	762.603	2.767
CL (78:11/18:0)	157	123	269	214	157	362	0.73	0.211	0.211	283.274	762.603	2.767
CL (80:9/20:4)	440	287	544	366	307	460	1.20	0.608	0.608	303.244	778.611	2.797
CL (80:13/18:0)	225	169	287	233	195	261	0.97	0.500	0.500	283.274	774.581	2.797
CL (82:4/16:0)	161	119	230	204	97	261	0.79	0.500	0.500	255.242	797.688	2.798
CL (82:4/18:1)	364	243	537	477	230	617	0.76	0.404	0.404	281.259	797.688	2.798
CL (82:6/16:0)	555	353	777	763	383	1007	0.73	0.282	0.282	255.242	795.608	2.798
CL (82:6/18:1)	995	665	1519	1381	647	1928	0.72	0.320	0.320	281.259	795.608	2.798
CL (82:6/18:2)	360	265	753	802	388	951	0.45	0.054	0.054	279.243	795.608	2.798
CL (82:8/16:0)	1044	845	1432	1334	982	1461	0.78	0.168	0.168	255.242	793.644	2.798
CL (82:8/18:2)	1009	730	2330	2294	1016	2703	0.44	0.089	0.089	279.243	793.644	2.798
CL (82:9/20:4)	298	229	467	267	239	339	1.12	0.320	0.320	303.244	792.586	2.798
CL (82:10/16:0)	247	222	300	236	229	299	1.05	0.736	0.736	255.242	791.609	1.679
CL (82:10/16:1)	322	236	409	368	256	518	0.88	0.608	0.608	253.226	791.607	2.798
CL (82:10/18:1)	253	227	420	316	259	353	0.80	0.500	0.500	381.258	791.609	2.798
CL (82:11/18:0)	234	180	430	344	243	466	0.68	0.188	0.188	283.273	790.640	2.798
CL (82:11/22:6)	282	206	452	352	277	525	0.80	0.211	0.211	327.245	790.640	2.798
CL (82:13/18:0)	373	196	598	361	152	553	1.03	0.822	0.822	283.274	788.613	2.798
CL (82:13/18:1)	296	225	388	260	175	340	1.14	0.404	0.404	281.259	788.613	2.798
CL (84:11/16:0)	0	0	0	0	0	0	NC	0.457	0.458	255.242	804.616	2.802
CL (84:13/16:0)	277	157	369	275	181	453	1.01	0.797	0.797	255.242	802.652	2.801
CL (84:15/20:4)	185	115	341	181	134	230	1.02	0.847	0.847	303.244	800.702	2.801
CL (86:10/16:0)	0	0	867	0	0	0	NC	0.095	0.095	255.243	819.674	2.805
CL (86:10/18:1)	0	0	2316	0	0	0	NC	0.086	0.086	281.259	819.674	2.805
CL (86:11/16:0)	3380	0	6950	2445	0	3938	1.38	0.237	0.237	255.242	818.668	2.804
CL (86:12/16:0)	1573	1011	3550	2799	1563	3575	0.56	0.116	0.116	255.242	817.690	2.804
CL (86:12/18:1)	130	83	293	161	103	274	0.81	0.724	0.724	281.258	817.690	2.804
CL (86:12/18:2)	4825	2538	9635	9066	5115	12005	0.53	0.123	0.124	279.243	817.690	2.804
CL (86:13/16:0)	4109	0	9619	5824	1659	9686	0.71	0.570	0.570	255.242	816.621	2.804

Lipid species	Carriers of pathogenic *LDLR* variant (n = 17)			Carriers with no detected pathogenic *LDLR* variant (n = 24)			Fold change	*p*-Value		Lipid annotation		
	Median	Q1	Q3	Median	Q1	Q3		Raw *p*-Value	FDR-adjusted *p*-Value	Scan m/z	m/z	Correction
CL (86:13/16:1)	127	0	293	89	25	149	1.43	0.527	0.527	253.226	816.621	2.804
CL (86:14/18:2)	145	92	225	178	123	199	0.81	0.847	0.847	279.243	815.700	2.804
CL (86:14/18:3)	133	76	201	152	103	193	0.87	0.748	0.748	277.226	815.700	2.804
CL (86:15/16:0)	334	268	450	393	284	508	0.85	0.304	0.305	255.242	814.669	2.804
CL (86:15/16:1)	200	158	241	192	159	212	1.04	0.531	0.532	253.226	814.669	2.804
CL (88:11/16:0)	0	0	114	0	0	63	NC	0.614	0.614	255.242	832.682	2.809
CL (88:11/18:1)	0	0	262	0	0	191	NC	0.502	0.502	281.259	832.682	2.809
CL (88:12/18:2)	369	198	665	411	234	636	0.90	0.873	0.873	279.243	831.681	2.809
CL (88:13/18:0)	88	0	167	156	94	271	0.56	0.116	0.116	283.274	830.680	2.809
CL (88:13/18:1)	119	0	173	182	108	224	0.65	0.123	0.123	281.259	830.680	2.809
CL (88:13/18:2)	453	0	800	907	634	1310	0.50	0.054	0.054	279.243	830.680	2.809
CL (88:14/18:2)	209	148	294	164	128	207	1.27	0.098	0.098	279.250	829.695	2.809
CL (88:15/18:2)	537	311	766	485	398	522	1.11	0.608	0.608	279.243	828.678	2.809
Diacylglycerol pyrophosphate (DGPP)												
DGPP (40:6/20:4)	844	671	1,179	662	553	777	1.28	0.018	0.181	303.244	827.677	1.664
DGPP (38:3/18:1)	0	0	0	0	0	0	NC	1.000	1.000	281.259	805.614	1.628
DGPP (38:4/16:0)	80	0	201	121	0	209	0.66	0.319	0.319	255.242	803.637	1.828
DGPP (38:4/18:2)	431	0	712	365	0	728	1.18	0.746	0.746	279.243	803.637	1.828
DGPP (38:5/18:2)	230	139	290	228	194	251	1.01	1.000	1.000	279.243	801.651	1.628
DGPP (40:5/20:3)	203	133	269	193	176	233	1.05	0.898	0.898	305.260	829.682	1.665
DGPP (40:7/20:4)	237	60	353	207	152	289	1.14	0.797	0.797	303.244	825.675	1.664
DGPP (O-36:1/18:0)	332	307	376	363	278	507	0.91	0.422	0.422	283.274	767.637	1.642
DGPP (O-40:4/20:4)	382	253	457	463	371	639	0.82	0.095	0.095	303.244	817.647	1.717
Dimethylphosphatidylethanolamine (DMPE)												
DMPE (32:2/18:2)	347	296	468	521	417	676	0.67	0.010	0.181	279.243	714.592	1.587
DMPE (34:2/16:0)	274	219	357	334	288	384	0.82	0.034	0.245	255.243	742.572	1.623
DMPE (34:2/18:2)	1,557	1,316	1,988	2,069	1,760	2,515	0.75	0.008	0.181	279.243	742.572	1.623
DMPE (O-44:2)	223	178	279	154	134	224	1.45	0.016	0.181	224.077	868.718	1.716
DMPE (32:1/16:0)	187	147	243	202	146	247	0.93	0.564	0.564	255.242	716.607	1.587
DMPE (32:1/18:1)	449	349	527	495	363	625	0.91	0.404	0.404	281.259	716.607	1.587
DMPE (34:1/18:0)	159	141	175	192	120	215	0.83	0.320	0.320	283.274	744.612	1.623
DMPE (34:1/18:1)	1080	903	1202	1188	890	1415	0.91	0.248	0.248	281.259	744.612	1.623
DMPE (34:2/18:1)	512	440	716	553	425	627	0.93	0.748	0.748	281.259	742.572	1.623
DMPE (34:3/18:2)	360	273	427	364	314	497	0.99	0.700	0.700	279.243	740.546	1.623
DMPE (34:4/20:4)	660	597	747	841	576	943	0.79	0.132	0.132	303.244	738.588	1.622
DMPE (34:5/20:4)	268	167	369	219	180	243	1.22	0.336	0.336	303.244	736.540	1.622
DMPE (36:2/18:2)	393	344	538	466	435	517	0.84	0.109	0.109	279.243	770.654	1.660

Lipid species	Carriers of pathogenic *LDLR* variant (n = 17)			Carriers with no detected pathogenic *LDLR* variant (n = 24)			Fold change	*p*-Value		Lipid annotation		
	Median	Q1	Q3	Median	Q1	Q3		Raw *p*-Value	FDR-adjusted *p*-Value	Scan m/z	m/z	Correction
DMPE (36:3/16:0)	397	256	588	451	311	599	0.88	0.653	0.653	255.242	768.587	1.659
DMPE (36:4/16:0)	330	253	383	335	260	378	0.98	0.923	0.923	255.242	766.573	1.659
DMPE (36:4/16:1)	164	108	271	211	110	360	0.78	0.422	0.422	253.226	766.573	1.659
DMPE (36:5/14:0)	190	172	341	265	160	320	0.72	0.949	0.949	227.210	764.594	1.659
DMPE (36:5/16:0)	329	278	382	340	256	423	0.97	0.773	0.773	255.242	764.594	1.659
DMPE (38:3/16:0)	559	376	821	756	351	998	0.74	0.320	0.320	255.242	796.672	1.697
DMPE (38:3/18:1)	1456	906	2081	1966	885	2511	0.74	0.352	0.352	281.259	796.672	1.697
DMPE (38:4/16:0)	1977	1405	2992	3061	1300	3913	0.65	0.275	0.275	255.242	794.592	1.697
DMPE (38:4/18:1)	4139	2684	6134	5745	2530	7777	0.72	0.441	0.441	281.259	794.592	1.697
DMPE (38:4/18:2)	1494	1065	3075	3502	1501	3916	0.43	0.063	0.063	279.243	794.592	1.697
DMPE (38:5/16:0)	3494	2758	4998	4400	3364	5206	0.79	0.199	0.199	255.242	792.586	1.697
DMPE (38:5/18:1)	235	181	274	222	190	278	1.06	0.748	0.748	281.259	792.586	1.697
DMPE (38:5/18:2)	3829	3073	8907	9696	3896	11185	0.39	0.083	0.083	279.243	792.586	1.697
DMPE (38:6/16:0)	598	524	678	643	527	850	0.93	0.797	0.797	255.242	790.640	1.696
DMPE (38:6/16:1)	1086	765	1224	1139	741	1667	0.95	0.949	0.949	253.226	790.640	1.696
DMPE (38:6/18:1)	394	335	453	428	342	465	0.92	0.653	0.653	281.259	790.640	1.696
DMPE (38:6/20:4)	146	112	226	139	112	199	1.05	0.936	0.936	303.244	790.640	1.696
DMPE (38:7/18:2)	273	202	389	306	270	354	0.89	0.422	0.422	279.243	788.638	1.696
DMPE (40:4/18:0)	566	316	735	570	312	831	0.99	0.898	0.898	283.274	822.627	1.736
DMPE (40:5/18:0)	1195	1004	1595	1411	1079	1796	0.85	0.275	0.275	283.274	820.635	1.735
DMPE (40:5/18:1)	1998	1383	2253	1962	1166	2283	1.02	0.607	0.607	281.258	820.651	1.735
DMPE (40:5/18:2)	0	0	0	0	0	0	NC	0.430	0.430	279.243	820.635	1.735
DMPE (40:5/20:3)	286	222	467	478	264	632	0.60	0.158	0.158	305.260	820.619	1.735
DMPE (40:6)	459	418	526	485	431	520	0.95	0.422	0.422	305.260	818.652	1.735
DMPE (40:6/18:1)	35476	28870	38773	37124	29911	40729	0.96	0.700	0.700	281.259	818.652	1.735
DMPE (40:6/18:2)	1699	1202	2457	1294	941	2517	1.31	0.352	0.352	279.243	818.652	1.735
DMPE (40:6/20:3)	695	594	1321	1291	686	1915	0.54	0.168	0.168	305.260	818.652	1.735
DMPE (40:6/20:4)	764	435	1097	924	337	1149	0.83	0.773	0.773	303.244	818.652	1.735
DMPE (40:7)	607	430	700	647	583	723	0.94	0.248	0.248	331.275	816.633	1.735
DMPE (40:7/18:1)	877	621	1130	727	577	827	1.21	0.070	0.070	281.259	816.633	1.735
DMPE (40:7/18:2)	41650	31212	67499	50095	40164	61637	0.83	0.461	0.461	279.243	816.633	1.735
DMPE (40:7/20:4)	3087	1964	4896	3111	2083	4396	0.99	0.923	0.923	303.245	816.633	1.735
DMPE (40:8/18:2)	658	440	784	613	507	723	1.07	0.700	0.700	279.243	814.669	1.734
DMPE (40:8/18:3)	488	293	670	552	324	674	0.88	0.677	0.677	277.227	814.669	1.734
DMPE (40:8/20:5)	207	139	274	286	132	480	0.72	0.261	0.261	301.228	814.669	1.734
DMPE (42:6/20:3)	0	0	495	141	0	428	0.00	0.595	0.595	305.259	846.652	1.774
DMPE (42:7)	323	268	352	325	307	365	0.99	0.665	0.665	225.642	844.694	1.774
DMPE (42:7/20:2)	472	405	625	606	466	726	0.78	0.235	0.235	307.274	844.694	1.774
DMPE (42:7/20:3)	485	350	592	500	458	568	0.97	0.564	0.564	305.259	844.694	1.774

Lipid species	Carriers of pathogenic *LDLR* variant (n = 17)			Carriers with no detected pathogenic *LDLR* variant (n = 24)			Fold change	*p*-Value		Lipid annotation		
	Median	Q1	Q3	Median	Q1	Q3		Raw *p*-Value	FDR-adjusted *p*-Value	Scan m/z	m/z	Correction
DMPE (42:8/20:3)	7063	4715	7831	7257	5735	9134	0.97	0.261	0.261	305.259	842.692	1.774
DMPE (42:8/20:4)	967	0	1812	1171	391	1346	0.83	0.923	0.923	303.244	842.692	1.774
DMPE (42:8/22:5)	240	132	336	266	161	359	0.90	0.653	0.653	329.259	842.692	1.774
DMPE (42:8/22:6)	185	101	264	198	154	329	0.93	0.235	0.235	327.244	842.692	1.774
DMPE (O-24:6)	247	219	283	241	202	264	1.03	0.653	0.653	225.642	566.416	1.443
DMPE (O-42:2)	441	400	516	348	316	468	1.27	0.098	0.098	225.642	840.690	1.678
DMPE (O-42:6/19:1)	192	65	234	276	157	367	0.69	0.078	0.078	295.238	832.644	1.675
Lysodimethylphosphatidylethanolamine (LDMPE)												
LDMPE (22:5)	725	626	816	825	615	940	0.88	0.386	0.386	225.642	568.418	1.417
LDMPE (24:5)	303	264	347	289	246	326	1.05	0.724	0.724	225.642	596.446	1.450
LDMPE (O-24:0)	221	189	326	277	158	323	0.80	0.700	0.700	225.642	578.428	1.418
Lysophosphatidylcholine (LPC)												
LPC (38:4)[a]	269	235	365	198	168	269	1.36	0.013	0.181	168.049	868.718	1.716
LPC (38:4)[b]	223	178	279	154	134	224	1.45	0.016	0.181	224.077	868.718	1.716
LPC (O-32:8)	1,375	1,163	1,473	1,169	1,008	1,271	1.18	0.043	0.281	168.049	776.589	1.603
LPC (16:0)[a]	188	166	216	227	159	269	0.83	0.275	0.275	224.078	554.404	1.338
LPC (16:0)[b]	725	626	816	825	615	940	0.88	0.386	0.386	224.078	554.404	1.338
LPC (18:0)	303	264	347	289	246	326	1.05	0.724	0.724	224.078	582.432	1.368
LPC (18:1)	247	219	283	241	202	264	1.03	0.653	0.653	224.079	580.430	1.368
LPC (18:2)	221	189	326	277	158	323	0.80	0.700	0.700	168.049	578.428	1.368
LPC (34:2)[a]	607	430	700	647	583	723	0.94	0.248	0.248	168.049	816.621	1.641
LPC (34:2)[b]	858	631	959	869	790	961	0.99	0.564	0.564	168.049	816.621	1.641
LPC (38:3)	261	195	292	237	202	263	1.10	0.369	0.369	224.078	870.720	1.716
LPC (O-30:6)	190	138	379	238	140	299	0.80	0.773	0.773	168.047	724.559	1.561
LPC (O-34:6)	172	121	237	214	140	302	0.80	0.282	0.282	168.048	794.592	1.636
LPC (O-34:7)	210	177	288	279	210	309	0.75	0.205	0.205	224.077	792.586	1.636
LPC (O-34:8)	289	251	363	279	242	335	1.04	0.413	0.413	168.050	790.640	1.636
LPC (O-36:8)[a]	459	418	526	485	431	520	0.95	0.422	0.422	168.049	818.652	1.673
LPC (O-36:8)[b]	763	681	810	783	685	807	0.97	0.949	0.949	168.049	818.652	1.673
Monomethylphosphatidylethanolamine (MMPE)												
MMPE (36:5/16:0)	168	129	216	249	192	371	0.67	0.002	0.181	255.243	750.577	1.640
MMPE (38:6/18:1)	168	127	222	225	177	342	0.75	0.012	0.181	281.259	776.589	1.677
MMPE (32:3/18:2)	427	302	691	539	413	680	0.79	0.369	0.369	279.243	698.524	1.569
MMPE (34:3/18:2)	589	495	1000	796	550	1031	0.74	0.422	0.422	279.243	726.576	1.604
MMPE (34:4/18:2)	350	301	541	405	348	494	0.87	0.461	0.461	279.243	724.559	1.604
MMPE (34:4/20:4)	335	200	439	276	187	430	1.22	0.773	0.773	303.244	724.559	1.604
MMPE (34:5/20:4)	2502	1734	3682	2475	1903	2882	1.01	0.748	0.748	303.244	722.541	1.604
MMPE (36:4/20:3)	311	227	461	325	227	353	0.96	0.688	0.688	305.258	752.594	1.641
MMPE (36:5/20:4)	4357	2792	6710	3729	3288	4961	1.17	0.404	0.404	303.244	750.577	1.640

Lipid species	Carriers of pathogenic *LDLR* variant (n = 17)			Carriers with no detected pathogenic *LDLR* variant (n = 24)			Fold change	*p*-Value		Lipid annotation		
	Median	Q1	Q3	Median	Q1	Q3		Raw *p*-Value	FDR-adjusted *p*-Value	Scan m/z	m/z	Correction
MMPE (36:6/20:4)	1803	1594	2954	1844	1269	2279	0.98	0.290	0.290	303.244	748.561	1.640
MMPE (36:6/20:5)	178	112	318	218	158	285	0.82	0.275	0.275	301.227	748.561	1.640
MMPE (36:7/22:6)	391	197	455	387	281	431	1.01	0.677	0.677	327.244	746.587	1.640
MMPE (38:5/16:0)	475	426	540	519	434	562	0.91	0.596	0.597	255.242	778.595	1.678
MMPE (38:5/18:2)	108	89	299	210	110	278	0.51	0.521	0.521	279.243	778.595	1.678
MMPE (38:5/22:4)	354	193	575	335	219	410	1.06	0.630	0.630	331.275	778.595	1.678
MMPE (38:6/16:0)	375	332	428	403	331	513	0.93	0.211	0.211	255.242	776.589	1.677
MMPE (38:6/22:5)	384	270	482	395	280	470	0.97	0.949	0.949	329.259	776.589	1.677
MMPE (38:7/22:6)	433	308	538	413	349	494	1.05	0.923	0.923	327.244	774.630	1.677
MMPE (38:8/22:6)	226	179	258	230	159	260	0.98	0.608	0.608	327.244	772.618	1.677
MMPE (40:5/18:1)	266	229	281	249	193	296	1.07	0.773	0.773	281.259	806.634	1.716
MMPE (40:6/18:1)	2004	1759	2168	1964	1764	2387	1.02	0.677	0.677	281.259	804.623	1.715
MMPE (40:6/18:2)	272	134	365	230	102	357	1.18	0.585	0.585	279.243	804.623	1.715
MMPE (40:7/18:1)	464	386	549	440	368	503	1.05	0.822	0.822	281.259	802.652	1.715
MMPE (40:7/18:2)	2391	1704	3418	2896	2316	3311	0.83	0.352	0.352	279.243	802.652	1.715
MMPE (40:7/20:4)	325	166	512	309	190	386	1.05	0.665	0.665	303.244	802.652	1.715
MMPE (40:8/18:2)	810	609	1010	672	629	927	1.20	0.847	0.847	279.243	800.702	1.715
MMPE (42:7/20:4)	91	0	216	114	33	239	0.80	0.771	0.771	303.244	830.630	1.754
MMPE (42:8/20:3)	571	449	656	591	479	728	0.97	0.422	0.422	305.259	828.678	1.754
MMPE (42:8/20:4)	467	334	517	386	333	435	1.21	0.158	0.158	303.244	828.678	1.754
MMPE (O-38:6/22:6)	275	231	499	364	290	646	0.75	0.188	0.188	327.245	762.612	1.674
MMPE (O-40:5/22:5)	181	156	253	198	166	242	0.92	0.490	0.490	329.259	792.586	1.712
				N-acyl phosphatidylethanolamine (NAPE)								
NAPE (48:6/18:1)	165	140	236	232	175	283	0.71	0.032	0.238	277.190	916.766	1.860
NAPE (54:0/16:0)	235	169	322	371	265	484	0.63	0.006	0.181	255.241	1012.862	1.993
NAPE (56:0/16:0)	157	141	239	286	181	376	0.55	0.018	0.181	255.243	1040.846	2.038
NAPE (56:0/18:0)	111	77	149	171	128	209	0.65	0.002	0.181	283.273	1040.846	2.038
NAPE (56:1/16:0)	138	120	225	255	160	353	0.54	0.007	0.181	255.241	1038.833	2.038
NAPE (56:1/18:1)	258	207	446	474	344	766	0.54	0.018	0.181	281.258	1038.833	2.038
NAPE (56:2/18:2)	152	101	206	209	190	338	0.73	0.014	0.181	279.244	1036.886	2.037
NAPE (58:1/18:1)	178	141	254	261	165	407	0.68	0.037	0.249	281.258	1066.871	2.084
NAPE (58:2/18:1)	107	92	191	217	110	284	0.49	0.034	0.245	281.258	1064.903	2.084
NAPE (42:1/16:0)	2609	1933	3025	2631	2215	3109	0.99	0.480	0.480	255.242	842.663	1.741
NAPE (42:1/18:1)	1961	1285	2466	1734	1476	2087	1.13	0.521	0.521	281.259	842.663	1.741
NAPE (44:1/18:0)	1142	958	1235	1127	923	1378	1.01	0.773	0.773	283.274	870.720	1.780
NAPE (52:11/18:1)	84	63	141	154	72	359	0.55	0.109	0.109	281.259	962.796	1.944
NAPE (56:2/16:0)	166	128	287	234	173	296	0.71	0.078	0.078	255.242	36.886	2.037
NAPE (58:2/18:2)	117	83	146	152	113	231	0.77	0.083	0.083	279.242	1064.903	2.084

Lipid species	Carriers of pathogenic *LDLR* variant (n = 17)			Carriers with no detected pathogenic *LDLR* variant (n = 24)			Fold change	*p*-Value		Lipid annotation		
	Median	Q1	Q3	Median	Q1	Q3		Raw *p*-Value	FDR-adjusted *p*-Value	Scan m/z	m/z	Correction
NAPE (58:3/18:1)	173	135	384	264	180	378	0.66	0.275	0.275	281.258	62.912	2.083
Phosphatidic acid (PA)												
PA (36:2/18:2)	111	79	189	142	105	181	0.78	0.352	0.352	279.243	609.560	1.581
PA (38:4/20:4)	551	370	790	494	366	687	1.12	0.822	0.822	303.244	723.549	1.616
Phosphatidylcholine (PC)												
PC (36:0/16:0)	126	103	163	179	133	244	0.70	0.009	0.181	255.242	848.656	1.679
PC (36:5/20:4)	495	432	572	335	300	476	1.48	0.002	0.181	303.244	838.633	1.678
PC (38:4/18:0)	3,974	3,577	5,064	2,595	2,434	4,548	1.53	0.013	0.181	283.275	868.718	1.716
PC (38:4/20:4)	13,369	11,268	17,664	7,679	6,976	14,681	1.74	0.016	0.181	303.244	868.718	1.716
PC (38:4/22:4)	401	329	440	289	259	333	1.39	0.011	0.181	331.275	868.718	1.716
PC (38:5/18:1)	947	697	1,152	635	481	849	1.49	0.008	0.181	281.259	866.716	1.716
PC (38:5/20:4)	2,372	1,690	3,124	1,580	1,094	2,261	1.50	0.009	0.181	303.244	866.716	1.716
PC (O-36:1/19:1)	267	192	360	367	235	478	0.73	0.043	0.281	295.238	832.644	1.675
PC (O-36:2/18:1)	441	367	482	348	318	412	1.27	0.034	0.245	281.259	830.630	1.675
PC (O-36:5/20:5)	0	0	84	93	46	227	0.00	0.031	0.238	301.228	824.647	1.674
PC (O-38:4/20:4)	1,639	1,509	2,098	1,174	1,041	1,786	1.40	0.009	0.181	303.244	854.704	1.712
PC (O-38:5/20:4)	1,185	917	1,363	602	333	1,197	1.97	0.019	0.181	303.244	852.702	1.712
PC (30:0/14:0)	190	172	341	265	160	320	0.72	0.949	0.949	227.210	764.598	1.570
PC (32:0/16:0)	3494	2758	4998	4400	3364	5206	0.79	0.199	0.199	255.242	792.586	1.605
PC (32:1/16:0)	598	524	678	643	527	850	0.93	0.797	0.797	255.242	790.640	1.605
PC (32:1/16:1)	1086	765	1224	1139	741	1667	0.95	0.949	0.949	253.226	790.640	1.605
PC (32:1/18:1)	394	335	453	428	342	465	0.92	0.653	0.653	281.259	790.640	1.605
PC (32:2/18:2)	273	202	389	306	270	354	0.89	0.422	0.422	279.243	788.606	1.605
PC (34:0/16:0)	989	883	1102	1056	925	1157	0.94	0.585	0.585	255.242	820.619	1.642
PC (34:0/18:0)	1195	1004	1595	1411	1079	1796	0.85	0.275	0.275	283.274	820.619	1.642
PC (34:1/16:0)	10935	9665	11570	11733	9352	12496	0.93	0.542	0.542	255.243	818.652	1.642
PC (34:1/16:1)	136	103	196	136	105	181	1.00	0.835	0.835	253.226	818.652	1.642
PC (34:1/18:1)	35476	28870	38773	37124	29911	40729	0.96	0.700	0.700	281.259	818.652	1.642
PC (34:2/16:0)	13378	9853	17747	15729	14125	18080	0.85	0.290	0.290	255.244	816.621	1.641
PC (34:2/16:1)	336	281	470	287	230	340	1.17	0.116	0.116	253.227	816.621	1.641
PC (34:2/18:1)	877	621	1130	727	577	827	1.21	0.070	0.070	281.259	816.621	1.641
PC (34:2/18:2)	48669	32968	67499	58789	50095	68478	0.83	0.223	0.223	279.245	816.621	1.641
PC (34:3/16:0)	334	268	450	393	284	508	0.85	0.304	0.305	255.242	814.619	1.641
PC (34:3/16:1)	200	158	241	192	159	212	1.04	0.531	0.532	253.226	814.619	1.641
PC (34:3/18:2)	658	440	784	613	507	723	1.07	0.700	0.700	279.243	814.619	1.641
PC (34:3/18:3)	499	416	959	645	505	787	0.77	0.521	0.521	277.227	814.619	1.641

Lipid species	Carriers of pathogenic *LDLR* variant (n = 17)			Carriers with no detected pathogenic *LDLR* variant (n = 24)			Fold change	*p*-Value		Lipid annotation		
	Median	Q1	Q3	Median	Q1	Q3		Raw *p*-Value	FDR-adjusted *p*-Value	Scan m/z	m/z	Correction
PC (34:4/20:4)	278	210	356	241	198	299	1.16	0.173	0.173	303.244	812.701	1.641
PC (36:0/18:0)	254	236	314	270	243	300	0.94	0.712	0.712	283.273	848.656	1.679
PC (36:1)	365	319	423	361	321	399	1.01	0.470	0.470	168.049	846.696	1.679
PC (36:1/18:0)	2031	1807	2132	1946	1732	2050	1.04	0.422	0.422	283.274	846.696	1.679
PC (36:1/18:1)	4490	3648	4890	3905	3443	4716	1.15	0.404	0.404	281.259	846.696	1.679
PC (36:2)[a]	323	268	352	325	307	365	0.99	0.665	0.665	224.078	844.694	1.679
PC (36:2)[b]	603	487	651	579	510	639	1.04	1.000	1.000	224.078	844.694	1.679
PC (36:2/16:0)	396	336	421	412	374	459	0.96	0.188	0.188	255.242	844.694	1.679
PC (36:2/18:0)	5933	4876	7779	6405	5627	7277	0.93	1.000	1.000	283.275	844.694	1.679
PC (36:2/18:1)	3449	3013	4439	2642	2235	3688	1.31	0.158	0.158	281.259	844.694	1.679
PC (36:2/18:2)	19413	14488	25618	20314	17616	26378	0.96	0.564	0.564	279.243	844.694	1.679
PC (36:2/20:2)	472	405	625	606	466	726	0.78	0.235	0.235	307.274	844.694	1.679
PC (36:3)	320	268	383	306	276	338	1.05	0.542	0.542	224.078	842.604	1.678
PC (36:3/16:0)	2609	1933	3025	2631	2215	3109	0.99	0.480	0.480	255.242	842.604	1.678
PC (36:3/18:1)	1961	1285	2466	1734	1476	2087	1.13	0.521	0.521	281.259	842.604	1.678
PC (36:3/18:2)	4654	3134	6265	4178	3638	5086	1.11	0.773	0.773	279.243	842.604	1.678
PC (36:3/18:3)	190	123	285	227	155	248	0.84	0.724	0.724	277.227	842.604	1.678
PC (36:3/20:3)	7063	4715	7831	7257	5735	9134	0.97	0.261	0.261	305.259	842.604	1.678
PC (36:4)[a]	441	400	516	348	316	468	1.27	0.098	0.098	168.049	840.665	1.678
PC (36:4)[b]	531	423	594	440	371	588	1.21	0.116	0.116	168.049	840.665	1.678
PC (36:4/16:0)	8704	6613	10122	6397	5436	9485	1.36	0.149	0.149	255.243	840.665	1.678
PC (36:4/18:2)	1544	675	2044	1296	1071	1607	1.19	0.898	0.898	279.243	840.665	1.678
PC (36:4/20:4)	28840	22018	36985	20746	17789	32819	1.39	0.095	0.095	303.245	840.665	1.678
PC (36:5/16:0)	467	358	920	661	463	1094	0.71	0.328	0.328	255.242	838.633	1.678
PC (36:5/20:5)	1066	780	2038	1483	988	2572	0.72	0.542	0.542	301.228	838.633	1.678
PC (38:2)	382	343	428	370	266	414	1.03	0.608	0.608	224.079	872.718	1.717
PC (38:2/18:2)	192	153	232	206	184	231	0.93	0.320	0.320	279.243	872.718	1.717
PC (38:2/20:2)	238	190	259	220	176	295	1.08	0.847	0.847	307.274	872.718	1.717
PC (38:3/18:0)	1142	958	1235	1127	923	1378	1.01	0.773	0.773	283.275	870.720	1.716
PC (38:3/18:2)	149	105	207	167	148	199	0.89	0.451	0.451	279.243	870.720	1.716
PC (38:3/20:3)	2695	2034	3552	2793	2142	3799	0.97	0.724	0.724	305.260	870.720	1.716
PC (38:4/18:1)	305	250	368	294	227	331	1.04	0.163	0.163	281.259	868.718	1.716
PC (38:4/20:3)	624	479	841	585	439	695	1.07	0.275	0.275	305.259	868.718	1.716
PC (38:5/16:0)	672	507	775	636	519	734	1.06	0.923	0.923	255.242	866.716	1.716
PC (38:5/18:0)	284	207	504	326	205	485	0.87	0.835	0.835	283.274	866.716	1.716
PC (38:5/20:5)	601	446	1126	744	462	1239	0.81	0.630	0.630	301.228	866.716	1.716
PC (38:5/22:5)	1489	1085	1628	1320	1005	1576	1.13	0.521	0.521	329.259	866.716	1.716

Lipid species	Carriers of pathogenic *LDLR* variant (n = 17)			Carriers with no detected pathogenic *LDLR* variant (n = 24)			Fold change	*p*-Value		Lipid annotation		
	Median	Q1	Q3	Median	Q1	Q3		Raw *p*-Value	FDR-adjusted *p*-Value	Scan m/z	m/z	Correction
PC (38:6/16:0)	1514	1204	1995	1845	1255	2538	0.82	0.369	0.369	255.242	864.701	1.715
PC (38:6/18:2)	269	174	304	199	168	252	1.35	0.261	0.261	279.242	864.701	1.715
PC (38:6/20:4)	461	326	507	353	298	463	1.31	0.089	0.089	303.244	864.701	1.715
PC (38:6/20:5)	166	105	202	152	96	243	1.09	0.700	0.700	301.227	864.701	1.715
PC (38:6/22:6)	2881	2218	3744	3308	2301	4716	0.87	0.369	0.369	327.244	864.701	1.715
PC (40:5/18:0)	234	211	276	240	173	302	0.97	0.974	0.974	283.274	894.744	1.755
PC (40:5/22:5)	519	426	599	452	366	574	1.15	0.630	0.630	329.259	894.744	1.755
PC (40:6/18:0)	553	430	661	602	439	816	0.92	0.585	0.585	283.274	892.760	1.755
PC (40:6/22:6)	1021	765	1194	1100	767	1648	0.93	0.608	0.608	327.245	892.760	1.755
PC (40:7/22:6)	240	185	311	201	134	259	1.19	0.188	0.188	327.244	890.710	1.754
PC (O-32:0/16:0)	475	426	540	519	434	562	0.91	0.596	0.597	255.242	778.595	1.602
PC (O-34:1/16:0)	908	839	1065	900	842	999	1.01	0.949	0.949	255.243	804.619	1.638
PC (O-34:1/18:1)	2004	1759	2168	1964	1764	2387	1.02	0.653	0.653	281.259	804.619	1.638
PC (O-34:2/18:2)	2725	2233	3418	3092	2452	3432	0.88	0.320	0.320	279.243	802.652	1.638
PC (O-36:1/16:0)	253	202	293	308	215	400	0.82	0.255	0.255	255.242	832.644	1.675
PC (O-36:1/18:1)	805	698	957	720	632	925	1.12	0.336	0.336	281.259	832.644	1.675
PC (O-36:2/17:0)	245	199	263	235	187	288	1.04	0.873	0.873	269.258	830.630	1.675
PC (O-36:2/18:2)	1884	1400	2365	1974	1882	2052	0.95	0.542	0.542	279.243	830.630	1.675
PC (O-36:3/18:2)	718	511	772	578	487	695	1.24	0.223	0.223	279.243	828.678	1.675
PC (O-36:3/20:3)	529	449	641	591	475	728	0.90	0.320	0.320	305.259	828.678	1.675
PC (O-36:4/20:4)	2645	2181	3289	2210	1862	2794	1.20	0.102	0.102	303.244	826.659	1.674
PC (O-38:1/19:1)	111	86	146	133	104	176	0.84	0.140	0.140	295.239	860.689	1.713
PC (O-38:2/18:2)	184	120	223	205	184	217	0.89	0.194	0.194	279.243	858.708	1.713
PC (O-38:3/20:3)	399	316	432	344	298	428	1.16	0.847	0.847	305.259	856.690	1.713
PC (O-38:6/22:6)	307	228	409	310	192	413	0.99	0.974	0.974	327.244	850.700	1.712
PC (O-40:6/22:6)	183	159	210	200	141	250	0.92	0.785	0.785	327.246	878.722	1.751
				Phosphatidylethanolamine (PE)								
PE (34:2/16:0)	130	114	175	198	159	238	0.65	0.007	0.181	255.243	714.592	1.587
PE (34:2/18:2)	347	296	468	521	417	676	0.67	0.010	0.181	279.243	714.592	1.587
PE (36:2/16:0)	184	113	274	293	258	349	0.63	0.014	0.181	255.243	742.572	1.623
PE (36:2/18:0)	290	234	333	377	338	515	0.77	0.012	0.181	283.274	742.572	1.623
PE (36:2/18:2)	1,557	1,316	1,988	2,069	1,760	2,515	0.75	0.008	0.181	279.243	742.572	1.623
PE (38:5/20:4)	725	608	930	598	422	725	1.21	0.045	0.284	303.244	764.598	1.659
PE (34:1/16:0)	187	147	243	202	146	247	0.93	0.564	0.564	255.242	835.655	1.674
PE (34:1/18:1)	449	349	527	495	363	625	0.91	0.404	0.404	281.258	835.655	1.674
PE (36:0/18:0)	218	140	273	219	160	261	0.99	0.564	0.564	283.252	774.630	1.660
PE (36:1/16:0)	163	133	245	222	139	247	0.74	0.289	0.290	255.245	772.596	1.660
PE (36:1/18:0)	159	141	175	192	120	215	0.83	0.320	0.320	283.274	772.596	1.660

Lipid species	Carriers of pathogenic *LDLR* variant (n = 17)			Carriers with no detected pathogenic *LDLR* variant (n = 24)			Fold change	*p*-Value		Lipid annotation		
	**Median**	**Q1**	**Q3**	**Median**	**Q1**	**Q3**		**Raw *p*-Value**	**FDR-adjusted *p*-Value**	**Scan m/z**	**m/z**	**Correction**
PE (36:1/18:1)	1080	903	1202	1188	890	1415	0.91	0.248	0.248	281.259	772.596	1.660
PE (36:2/18:1)	512	440	716	553	425	627	0.93	0.748	0.748	281.259	742.572	1.623
PE (36:3/18:2)	360	273	427	364	314	497	0.99	0.700	0.700	279.243	754.611	1.623
PE (36:4/16:0)	344	272	368	415	299	468	0.83	0.109	0.109	255.242	752.594	1.641
PE (36:4/20:4)	660	597	747	841	576	943	0.79	0.132	0.132	303.244	752.594	1.641
PE (36:5/20:4)	126	60	343	118	0	214	1.07	0.335	0.335	303.244	736.551	1.622
PE (38:2/18:2)	101	0	301	0	0	201	NC	0.904	0.905	279.243	770.659	1.660
PE (38:3/16:0)	0	0	238	0	0	181	NC	0.667	0.667	255.242	796.672	1.697
PE (38:3/20:3)	378	334	443	453	361	526	0.83	0.153	0.153	305.259	796.672	1.697
PE (38:4/16:0)	330	253	383	335	260	378	0.98	0.923	0.923	255.240	794.592	1.697
PE (38:4/18:0)	1146	1075	1420	1252	1037	1571	0.91	0.724	0.724	283.274	794.592	1.697
PE (38:4/20:4)	3092	2684	3693	3060	2803	4070	1.01	0.677	0.677	303.244	794.592	1.697
PE (38:5/16:0)	329	278	382	340	256	423	0.97	0.773	0.773	255.242	764.598	1.659
PE (38:5/18:1)	269	223	342	219	181	294	1.23	0.109	0.109	281.259	764.598	1.659
PE (38:5/20:5)	107	85	152	145	106	216	0.74	0.248	0.248	301.227	764.598	1.659
PE (40:3/18:1)	0	0	0	0	0	0	NC	0.789	0.789	281.259	755.635	1.640
PE (40:4/18:1)	0	0	1097	0	0	941	NC	0.553	0.553	281.259	808.656	1.716
PE (40:5/18:1)	235	181	274	222	190	278	1.06	0.724	0.724	281.258	820.651	1.735
PE (40:5/20:4)	298	225	467	265	202	334	1.13	0.386	0.386	303.247	820.631	1.735
PE (40:5/22:5)	181	156	253	198	166	242	0.92	0.490	0.490	329.261	820.641	1.735
PE (40:6/22:6)	282	206	452	352	277	525	0.80	0.211	0.211	327.244	804.623	1.735
PE (O-36:2/18:1)	266	102	323	130	0	168	2.05	0.244	0.244	281.259	728.587	1.619
PE (O-36:3/18:2)	543	485	1000	772	550	1031	0.70	0.290	0.290	279.243	726.576	1.619
PE (O-38:4/20:3)	100	0	182	118	0	139	0.84	0.594	0.594	305.259	780.597	1.693
PE (O-38:4/20:4)	433	330	583	348	321	472	1.25	0.378	0.378	303.244	780.597	1.693
PE (O-38:5/20:4)	3942	2792	6710	3453	2923	4717	1.14	0.653	0.653	303.244	750.577	1.655
PE (O-40:5/20:4)	0	0	175	0	0	117	NC	0.715	0.715	303.244	792.586	1.712
PE (O-40:5/22:4)	136	0	241	0	0	255	NC	0.364	0.364	331.274	792.586	1.712
PE (O-40:6/22:5)	126	0	392	143	75	179	0.88	0.698	0.698	329.259	776.589	1.693
PE (O-40:7/22:6)	307	213	433	300	237	412	1.03	0.822	0.822	327.244	774.630	1.693
Phosphatidylglycerol (PG)												
PG (40:6/18:0)	0	0	0	102	0	221	0.00	0.019	0.181	283.271	821.649	1.717
PG (40:8/18:2)	7,442	4,308	12,768	11,572	9,808	13,801	0.64	0.015	0.181	279.244	817.647	1.717
PG (34:2/18:1)	103	0	186	105	0	213	0.98	0.534	0.535	281.259	745.583	1.606
PG (34:3/18:2)	156	0	206	0	0	204	NC	0.641	0.641	279.243	779.130	1.606
PG (36:5/20:4)	805	686	913	823	710	1045	0.98	0.564	0.564	303.244	767.637	1.642
PG (38:4/18:1)	364	243	537	477	230	617	0.76	0.404	0.404	281.259	797.688	1.680
PG (38:5/16:0)	226	0	327	0	0	268	NC	0.410	0.410	255.242	795.671	1.679

Lipid species	Carriers of pathogenic *LDLR* variant (n = 17)			Carriers with no detected pathogenic *LDLR* variant (n = 24)			Fold change	*p*-Value		Lipid annotation		
	Median	Q1	Q3	Median	Q1	Q3		Raw *p*-Value	FDR-adjusted *p*-Value	Scan m/z	m/z	Correction
PG (38:5/18:1)	995	665	1519	1381	647	1928	0.72	0.320	0.320	281.259	795.671	1.679
PG (38:5/18:2)	354	251	713	684	388	842	0.52	0.095	0.095	279.242	795.671	1.679
PG (38:6/16:0)	427	268	842	633	512	1012	0.68	0.089	0.089	255.242	793.592	1.679
PG (38:6/18:2)	1009	730	2330	2263	1016	2703	0.45	0.089	0.089	279.243	793.592	1.679
PG (38:7/16:1)	322	236	409	368	256	518	0.88	0.608	0.608	253.227	791.609	2.758
PG (40:5/18:1)	150	48	298	82	0	175	1.83	0.155	0.155	281.258	823.654	1.718
PG (40:6/18:2)	68	0	540	97	0	407	0.70	0.684	0.684	279.242	821.671	1.717
PG (40:7/18:1)	7901	6181	8582	7940	5854	9009	1.00	0.923	0.923	281.259	819.646	1.717
PG (40:7/20:3)	209	174	368	385	188	496	0.54	0.183	0.183	305.259	819.646	1.717
PG (40:7/20:4)	279	208	440	261	207	405	1.07	0.885	0.885	303.244	819.646	1.717
PG (40:8/20:4)	776	517	1258	792	580	1104	0.98	0.822	0.822	303.245	817.647	1.717
PG (42:7/20:3)	95	24	153	80	0	184	1.20	0.771	0.771	305.259	847.703	1.756
PG (42:8/20:4)	562	252	838	416	258	672	1.35	0.369	0.369	303.244	845.695	1.756
Phosphatidylinositol (PI)												
PI (34:2/18:1)	104	0	187	100	68	229	1.04	0.858	0.858	381.258	833.650	1.673
PI (34:3/18:2)	214	0	257	223	0	294	0.96	0.599	0.599	279.243	831.620	1.673
PI (36:0/18:0)	311	260	399	352	266	471	0.88	0.461	0.461	283.274	865.750	1.712
PI (38:4)	664	621	866	718	643	861	0.92	0.677	0.677	153.000	885.735	1.750
PI (38:4/18:0)	851	799	974	841	749	1005	1.01	0.822	0.822	283.275	885.735	1.750
PI (38:4/20:4)	881	814	1113	956	863	1126	0.92	0.677	0.677	303.244	885.735	1.750
PI (O-34:1/18:1)	356	273	393	378	297	437	0.94	0.211	0.211	281.259	821.628	1.717
PI (O-34:2/18:2)	258	165	385	221	131	426	1.17	0.652	0.653	279.243	819.658	1.669
Phosphatidylinositol phosphate (PIP)												
PIP (38:6/16:0)	177	130	233	161	103	204	1.10	0.607	0.607	255.241	961.784	1.762
Phosphatidylinositol bisphosphate (PIP2)												
PIP2 (36:3/18:1)	0	0	44	29	0	201	0.00	0.110	0.110	281.258	509.350	1.736
Phosphatidylinositol trisphosphate (PIP3)												
PIP3 (30:1/14:0)	52	29	72	104	47	135	0.50	0.011	0.181	227.210	509.348	1.635
PIP3 (30:1/16:0)	0	0	47	83	0	206	0.00	0.013	0.181	255.241	509.350	1.635
PIP3 (30:1/16:1)	117	49	158	190	88	301	0.61	0.032	0.238	253.227	509.348	1.635
PIP3 (34:0/16:0)	170	52	275	336	149	528	0.51	0.025	0.218	255.242	538.388	1.711
PIP3 (34:1/16:0)	757	382	1,315	1,699	680	2,613	0.45	0.013	0.181	255.242	537.429	1.711
PIP3 (34:1/16:1)	52	28	100	109	55	163	0.47	0.016	0.181	253.226	537.429	1.711
PIP3 (34:1/18:0)	65	33	95	100	64	177	0.65	0.025	0.218	283.274	537.429	1.711
PIP3 (34:1/18:1)	1,432	689	2,535	3,259	1,343	5,331	0.44	0.010	0.181	281.259	537.429	1.711
PIP3 (34:2/16:0)	80	53	109	122	103	171	0.66	0.006	0.181	255.243	536.386	1.710

Lipid species	Carriers of pathogenic *LDLR* variant (n = 17)			Carriers with no detected pathogenic *LDLR* variant (n = 24)			Fold change	*p*-Value		Lipid annotation		
	Median	Q1	Q3	Median	Q1	Q3		Raw *p*-Value	FDR-adjusted *p*-Value	Scan m/z	m/z	Correction
PIP3 (34:2/18:1)	45	16	91	100	44	154	0.45	0.037	0.249	281.259	536.386	1.710
PIP3 (34:2/18:2)	65	46	112	165	70	199	0.39	0.019	0.181	279.244	536.386	1.710
PIP3 (38:1/18:0)	453	196	833	994	487	1,408	0.46	0.023	0.213	283.274	565.415	1.789
PIP3 (38:1/18:1)	0	0	0	0	0	659	NA	0.009	0.181	281.259	565.415	1.789
PIP3 (38:3/18:2)	0	0	85	263	0	430	0.00	0.026	0.221	279.242	563.413	1.789
PIP3 (38:2/18:1)	0	0	0	0	0	0	NC	0.141	0.141	281.259	564.414	1.789
PIP3 (38:3/18:1)	1644	293	3134	5877	1093	8075	0.28	0.052	0.052	281.260	563.413	1.789
PIP3 (38:4/18:1)	0	0	83	0	0	331	NC	0.299	0.299	281.259	562.412	1.788
PIP3 (38:7/18:3)	61	36	106	121	60	176	0.50	0.058	0.058	277.228	559.362	1.787
Phosphatidylserine (PS)												
PS (O-38:5/20:4)	458	377	642	352	280	424	1.30	0.032	0.238	303.244	794.592	1.682
PS (34:0/16:0)	333	259	593	431	350	708	0.77	0.199	0.199	255.242	538.388	1.613
PS (34:0/18:0)	157	123	269	214	157	362	0.73	0.211	0.211	283.275	538.388	1.613
PS (34:7/20:5)	72	0	126	45	0	285	1.59	0.946	0.946	301.227	748.559	1.610
PS (36:0/18:0)	234	180	430	344	243	466	0.68	0.188	0.188	283.275	552.402	1.750
PS (36:1/18:0)	373	196	598	357	152	553	1.04	0.822	0.822	283.274	551.428	1.750
PS (36:1/18:1)	296	225	388	260	175	340	1.14	0.404	0.404	281.258	551.428	1.750
PS (38:4/18:0)	449	253	792	437	198	654	1.03	0.712	0.712	283.274	810.659	1.686
PS (38:4/20:4)	343	268	546	317	249	400	1.08	0.413	0.413	303.244	810.659	1.686
PS (38:4/PS)	140	65	229	155	66	202	0.90	0.835	0.835	305.255	562.438	1.788
PS (38:6/16:0)	219	203	243	227	199	246	0.96	0.949	0.949	255.244	806.634	1.685
PS (38:6/18:1)	181	97	278	249	178	266	0.73	0.211	0.211	281.260	806.634	1.685
PS (38:6/18:2)	157	98	285	196	141	239	0.80	0.677	0.677	279.244	806.634	1.685
PS (38:7/18:2)	140	101	288	174	0	256	0.81	0.948	0.948	279.243	804.616	1.685
PS (40:7/18:2)	87	60	199	169	86	216	0.51	0.275	0.275	279.241	832.646	1.723
PS (40:7/20:4)	227	146	291	161	139	186	1.41	0.058	0.058	303.244	832.646	1.723
PS (40:8/20:4)	309	230	435	277	208	339	1.12	0.500	0.500	303.244	830.630	1.723
PS (O-36:1/18:0)	145	126	244	182	140	233	0.80	0.470	0.470	283.274	774.606	1.646
PS (O-36:7/22:6)	275	231	499	364	290	646	0.75	0.188	0.188	327.244	762.589	1.674
PS (O-40:4/20:4)	0	0	636	0	0	249	NC	0.665	0.665	303.242	824.642	1.720

The data are presented as median relative abundance, along with the first (Q1) and third (Q3) quartiles. The fold change was calculated as the ratio of median abundance in participants with a pathogenic *LDLR* variant to median abundance in participants with no detected pathogenic *LDLR* variant. Raw *p*-Values were obtained using two-sided Mann-Whitney U tests. False discovery rate (FDR)-adjusted *p*-Values were calculated for all 423 negatively ionized glycerophospholipid species that were tested. After FDR correction, none of the lipid species remained statistically significant. NC indicates that the fold change could not be calculated because the median abundance in the panel-negative group (the denominator) was 0.

Repeated lipid labels marked with superscript letters (a, b) represent distinct analytical features that are recorded separately in the source dataset.

LipidView software, version 1.2: "Scan m/z" denotes the m/z of the characteristic diagnostic scan channel used for the analytical feature. "Target m/z" denotes the m/z of the assigned precursor/molecular ion. "Correction" denotes the LipidView isotope correction factor used to correct for isotopic peak distribution.

FDR = false discovery rate; CL = cardiolipin; DGPP = diacylglycerol pyrophosphate; DMPE = dimethylphosphatidylethanolamine; LDMPE = lysodimethylphosphatidylethanolamine; LPC = lysophosphatidylcholine; MMPE = monomethylphosphatidylethanolamine; NAPE = N-acyl phosphatidylethanolamine; PA = phosphatidic acid; PC = phosphatidylcholine; PE = phosphatidylethanolamine; PG = phosphatidylglycerol; PI = phosphatidylinositol; PIP = phosphatidylinositol phosphate; PIP2 = phosphatidylinositol bisphosphate; PIP3 = phosphatidylinositol trisphosphate; PS = phosphatidylserine
