# Supplementary material for: Plasma Lipidomic Phenotype Associated with Pathogenic LDLR Variants in Familial Hypercholesterolemia
Source: Int J Mol Sci. 2026 Oct 11;27(20):9011. doi: 10.3390/ijms27209011 (PMC13705200; doi:10.3390/ijms27209011)
Supplement: Supplementary file 1 [file ijms-27-09011-s001.zip › Table S3.pdf]

Table S3. Comparison of positively ionized glycerophospholipid analytical feature relative abundances according to pathogenic *LDLR* variant status.

Lipid feature annotation	Carriers of pathogenic *LDLR* variant (n = 17)			Carriers with no detected pathogenic *LDLR* variant (n = 24)			Fold change	*p*-Value		Lipid annotation		
	Median	Q1	Q3	Median	Q1	Q3		Raw *p*-Value	FDR-adjusted *p*-Value	Scan m/z	m/z	Correction
					Phosphatidylcholine (PC)							
PC (34:2)[a]	3,793	2,444	4,022	4,411	3,755	4,885	0.86	0.006	0.441	104.114	758.620	1.641
PC (34:2)[b]	747,234	511,986	941,754	959,563	778,547	1,032,860	0.78	0.014	0.441	125.009	758.620	1.641
PC (38:4)	344,947	294,211	465,008	256,083	200,837	328,785	1.35	0.015	0.441	104.114	810.652	1.716
PC (34:4)	1,463	1,054	2,687	0	0	1,358	2.67	0.016	0.441	104.112	754.580	1.641
PC (44:2)	158	99	216	255	186	462	0.62	0.027	0.477	104.112	898.799	1.837
PC (O-38:5)[a]	35,637	33,386	39,147	31,117	27,230	34,877	1.15	0.034	0.497	104.112	794.633	1.712
PC (O-34:0)	1,129	0	3,374	0	0	1,075	3.38	0.042	0.497	104.112	748.607	1.638
PC (18:0)	4564	3200	6827	5776	4275	6984	0.79	0.246	0.733	184.086	524.397	1.368
PC (30:0)	12229	10502	13217	11393	9206	12690	1.07	0.610	0.888	184.085	706.555	1.570
PC (30:1)[a]	409	309	513	358	265	451	1.14	0.194	0.706	104.112	704.554	1.569
PC (30:1)[b]	1447	1142	1824	1703	1405	1941	0.85	0.259	0.733	184.086	704.554	1.569
PC (30:2)	293	233	357	309	267	471	0.95	0.430	0.804	184.086	702.552	1.569
PC (32:0)	8147	6800	9517	7786	6866	8856	1.05	0.526	0.862	184.086	720.507	1.602
PC (32:1)[a]	38831	35891	44090	40086	36984	46574	0.97	0.632	0.888	184.086	732.604	1.605
PC (32:1)[b]	553	453	649	541	485	618	1.02	0.722	0.907	104.112	732.604	1.605
PC (32:2)	717	568	824	629	562	726	1.14	0.272	0.733	184.085	730.567	1.605
PC (34:0)[a]	19279	17340	23226	19324	15584	21819	1.00	0.546	0.862	184.086	762.643	1.642
PC (34:0)[b]	319	306	380	324	216	377	0.98	0.769	0.910	104.113	762.643	1.642
PC (34:1)[a]	11661	10070	12873	10994	9797	11960	1.06	0.315	0.733	184.086	760.636	1.642
PC (34:1)[b]	425	317	533	343	275	472	1.24	0.386	0.781	104.114	760.636	1.642
PC (34:3)[a]	4929	2551	5883	3871	1534	4914	1.27	0.137	0.694	125.008	756.604	1.641
PC (34:3)[b]	18657	16308	22686	16268	12108	20934	1.15	0.286	0.733	184.086	756.604	1.641
PC (36:0)	13007	10894	14531	13175	10912	17596	0.99	0.449	0.817	184.086	790.644	1.679
PC (36:1)[a]	215	137	298	227	191	292	0.95	0.404	0.781	104.114	788.665	1.679
PC (36:1)[b]	5293	4135	8730	5820	4736	6758	0.91	0.816	0.943	184.087	788.665	1.679
PC (36:2)[a]	2303	1832	2815	2526	2200	2935	0.91	0.210	0.711	184.087	786.653	1.679
PC (36:2)[b]	311	167	557	406	312	593	0.77	0.272	0.733	104.114	786.653	1.679
PC (36:3)[a]	483	391	547	457	404	509	1.06	0.404	0.781	184.087	784.637	1.678
PC (36:3)[b]	837	735	966	804	728	894	1.04	0.506	0.862	104.112	784.637	1.678

Lipid feature annotation	Carriers of pathogenic *LDLR* variant (n = 17)			Carriers with no detected pathogenic *LDLR* variant (n = 24)			Fold change	*p*-Value		Lipid annotation		
	Median	Q1	Q3	Median	Q1	Q3		Raw *p*-Value	FDR-adjusted *p*-Value	Scan m/z	m/z	Correction
PC (36:4)	34433	29765	41633	31359	23205	34535	1.10	0.075	0.497	104.114	782.621	1.678
PC (36:5)[a]	765	673	864	944	746	1136	0.81	0.142	0.694	125.008	780.605	1.678
PC (36:5)[b]	72564	59472	84502	67676	58685	74766	1.07	0.300	0.733	184.087	780.605	1.678
PC (36:5)[c]	36225	31787	49136	45738	32927	70231	0.79	0.361	0.778	104.114	780.605	1.678
PC (38:0)	13999	12284	16919	15005	13751	18819	0.93	0.412	0.781	184.086	818.655	1.718
PC (38:1)	2576	2013	2831	2492	2149	2844	1.03	0.817	0.943	184.086	816.658	1.718
PC (38:2)[a]	4732	4051	5410	4380	4095	5162	1.08	0.914	0.959	104.112	814.677	1.718
PC (38:2)[b]	138156	120885	165177	130092	121169	154014	1.06	0.914	0.959	184.087	814.677	1.718
PC (38:2)[c]	68155	50502	75099	64329	55994	71441	1.06	1.000	1.000	184.087	814.677	1.718
PC (38:3)[a]	2777	2642	3176	3027	2668	3278	0.92	0.449	0.817	104.113	798.637	1.713
PC (38:3)[b]	6096	4532	6479	6121	5482	7124	1.00	0.526	0.862	184.086	798.637	1.713
PC (38:5)[a]	16874	14185	20761	21276	17658	23284	0.79	0.057	0.497	125.008	808.636	1.716
PC (38:5)[b]	159992	112182	182665	130844	109287	161454	1.22	0.061	0.497	104.112	808.636	1.716
PC (38:6)[a]	34091	28342	43252	42230	36016	48408	0.81	0.066	0.497	104.112	806.622	1.715
PC (38:6)[b]	612463	559243	676908	509829	415585	601979	1.20	0.080	0.497	184.087	806.622	1.715
PC (40:4)	15660	10390	19479	15962	11838	22345	0.98	0.654	0.893	184.086	838.688	1.755
PC (40:5)	14885	12706	19274	16927	12626	19725	0.88	0.506	0.862	184.086	836.681	1.755
PC (40:6)[a]	303	268	337	279	242	308	1.09	0.164	0.704	104.112	834.656	1.755
PC (40:6)[b]	11246	7890	14090	8608	7506	12480	1.31	0.315	0.733	184.086	834.656	1.755
PC (O-34:1)	13311	10687	16309	14665	12731	17106	0.91	0.300	0.733	184.086	746.554	1.638
PC (O-34:2)	1545	1276	1802	1573	1454	1731	0.98	0.567	0.866	184.086	744.629	1.638
PC (O-34:3)	13554	10021	15765	12236	11472	13875	1.11	0.841	0.947	184.086	742.623	1.637
PC (O-36:1)	587103	517372	663402	622188	550553	705194	0.94	0.395	0.781	184.086	774.640	1.675
PC (O-36:2)	19162	14703	21504	18058	15684	22064	1.06	0.938	0.959	184.086	772.635	1.675
PC (O-36:3)	501	440	594	523	454	681	0.96	0.395	0.781	184.086	770.650	1.675
PC (O-36:4)[a]	316	284	345	286	234	325	1.11	0.089	0.521	104.112	768.636	1.674
PC (O-36:4)[b]	321	177	392	239	93	336	1.34	0.164	0.704	184.086	768.636	1.674
PC (O-36:5)	276	228	323	302	255	341	0.91	0.222	0.732	184.086	766.612	1.674
PC (O-38:0)	183	70	222	129	78	192	1.42	0.353	0.778	184.084	804.654	1.714
PC (O-38:1)	776	634	838	786	640	897	0.99	0.745	0.910	184.086	802.652	1.713
PC (O-38:2)	16235	11714	20919	15037	13859	18713	1.08	0.361	0.778	184.086	800.683	1.713

Lipid feature annotation	Carriers of pathogenic *LDLR* variant (n = 17)			Carriers with no detected pathogenic *LDLR* variant (n = 24)			Fold change	*p*-Value		Lipid annotation		
	Median	Q1	Q3	Median	Q1	Q3		Raw *p*-Value	FDR-adjusted *p*-Value	Scan m/z	m/z	Correction
PC (O-38:3)[a]	288232	245515	338923	295373	270577	332162	0.98	0.506	0.862	104.113	798.637	1.713
PC (O-38:3)[b]	22595	20471	27632	23173	19353	29294	0.98	0.988	1.000	184.086	798.637	1.713
PC (O-38:4)[a]	5168	3815	5910	4332	3735	5288	1.19	0.080	0.497	104.112	796.652	1.712
PC (O-38:4)[b]	2781	2419	3479	2522	2011	2981	1.10	0.150	0.704	125.008	796.652	1.712
PC (O-38:5)[b]	5659	3704	6299	4295	1666	5620	1.32	0.061	0.497	104.112	794.633	1.712
PC (O-38:6)	707	628	779	671	626	771	1.05	0.546	0.862	184.086	792.635	1.712
PC (O-40:0)[a]	147	94	248	174	133	275	0.85	0.092	0.521	184.086	832.639	1.753
PC (O-40:0)[b]	4679	4139	5095	5625	4330	6770	0.83	0.142	0.694	125.008	832.639	1.753
PC (O-40:2)	1060	932	1313	1061	894	1221	1.00	0.722	0.907	184.086	828.678	1.735
PC (O-40:3)	457566	384891	561788	483545	431088	589726	0.95	0.272	0.733	184.086	826.676	1.717
PC (O-40:4)	438	361	530	428	354	509	1.02	0.557	0.866	184.086	824.674	1.735
PC (O-40:5)	591	468	723	635	541	784	0.93	0.395	0.781	184.086	822.658	1.735
PC (O-40:6)	1718	1547	2037	1748	1608	2072	0.98	0.654	0.893	184.086	820.666	1.735
Lysophosphatidylcholine (LPC)												
LPC (34:2)	22,109	15,033	28,767	28,718	22,530	30,967	0.77	0.015	0.441	125.009	758.620	1.641
LPC (38:4)[a]	9,763	8,853	13,585	7,741	6,128	9,916	1.26	0.021	0.477	125.008	810.652	1.716
LPC (38:4)[b]	1,683	1,529	2,322	1,277	1,031	1,673	1.32	0.025	0.477	181.037	810.652	1.716
LPC (O-32:1)	282	201	320	206	94	276	1.37	0.047	0.497	125.006	718.609	1.601
LPC (18:0)[a]	142144	117952	154665	171319	128099	213646	0.83	0.159	0.704	125.008	524.397	1.368
LPC (18:0)[b]	17608	15256	20332	18775	16700	21706	0.94	0.286	0.733	104.114	524.397	1.368
LPC (18:1)[a]	655	598	826	720	586	847	0.91	0.745	0.910	184.086	522.399	1.368
LPC (18:1)[b]	5400	3872	7274	5224	4436	6329	1.03	0.938	0.959	104.114	522.399	1.368
LPC (18:1)[c]	1922	1514	2646	1915	1651	2267	1.00	0.938	0.959	125.008	522.399	1.368
LPC (18:2)[a]	880	626	968	744	618	915	1.18	0.126	0.687	125.008	520.372	1.368
LPC (18:2)[b]	9701	7343	11364	8463	6437	9596	1.15	0.178	0.704	104.114	520.372	1.368
LPC (18:2)[c]	1445	1229	1961	1882	1485	2733	0.77	0.189	0.705	184.086	520.372	1.368
LPC (30:0)	9029	7783	10529	9164	8591	10287	0.99	0.506	0.862	125.007	706.555	1.570
LPC (30:1)	1319	1120	1568	1207	948	1350	1.09	0.199	0.707	125.008	704.554	1.569
LPC (30:2)	6471	5392	8193	7367	6380	8435	0.88	0.330	0.755	125.008	702.552	1.569
LPC (32:0)	653	460	867	692	526	961	0.94	0.654	0.893	125.006	720.507	1.602
LPC (32:1)	575	482	643	597	515	628	0.96	0.769	0.910	125.008	732.604	1.605

Lipid feature annotation	Carriers of pathogenic *LDLR* variant (n = 17)			Carriers with no detected pathogenic *LDLR* variant (n = 24)			Fold change	*p*-Value		Lipid annotation		
	Median	Q1	Q3	Median	Q1	Q3		Raw *p*-Value	FDR-adjusted *p*-Value	Scan m/z	m/z	Correction
LPC (32:2)	197	145	261	180	138	259	1.09	0.688	0.902	125.006	730.567	1.605
LPC (34:0)	584	479	676	614	536	699	0.95	0.588	0.879	125.008	762.643	1.642
LPC (34:1)	33857	32035	44112	29741	23188	36656	1.14	0.210	0.711	125.009	760.636	1.642
LPC (36:0)	317	166	388	267	129	434	1.19	0.516	0.862	125.008	790.644	1.679
LPC (36:1)	381	295	417	333	311	400	1.15	0.805	0.943	125.008	788.665	1.679
LPC (36:2)	1384	1244	1682	1199	981	1542	1.15	0.240	0.733	125.008	786.653	1.679
LPC (36:3)	41690	32309	53977	47822	39421	58610	0.87	0.395	0.781	125.008	784.637	1.678
LPC (38:0)	723	543	837	652	582	830	1.11	0.632	0.888	125.005	818.655	1.718
LPC (38:1)	39061	31733	48555	40323	34429	46320	0.97	0.699	0.902	125.008	816.658	1.718
LPC (38:2)	8768	7306	9433	8275	6718	9543	1.06	0.722	0.907	125.008	814.677	1.718
LPC (38:3)	19902	15074	25041	20213	18240	25969	0.98	0.486	0.862	125.006	798.637	1.713
LPC (38:6)	242	133	276	87	0	200	2.79	0.068	0.497	125.008	806.622	1.715
LPC (40:4)	339	296	371	338	271	375	1.00	0.938	0.959	125.008	838.688	1.755
LPC (40:5)	25206	20682	29891	23618	20527	27821	1.07	0.610	0.888	125.008	836.681	1.755
LPC (40:6)	1702	1179	2582	2157	1750	2639	0.79	0.178	0.704	125.008	834.656	1.755
LPC (O-30:1)[a]	6250	5183	7869	7808	6504	8593	0.80	0.066	0.497	184.086	676.540	1.562
LPC (O-30:1)[b]	1397	1315	1502	1281	1098	1412	1.09	0.080	0.497	125.008	676.540	1.562
LPC (O-34:1)	3853	3460	4524	3755	3449	4186	1.03	0.546	0.862	125.008	746.554	1.638
LPC (O-34:2)	216	193	256	184	161	249	1.17	0.567	0.866	125.008	744.629	1.638
LPC (O-34:3)	849	631	979	786	652	893	1.08	0.769	0.910	125.008	742.623	1.637
LPC (O-36:1)	12749	10838	14791	13203	11776	16176	0.97	0.546	0.862	125.008	774.640	1.675
LPC (O-36:2)	763	647	878	756	640	836	1.01	0.676	0.902	125.008	772.635	1.675
LPC (O-36:3)	259	160	361	233	199	276	1.11	0.676	0.902	125.006	770.650	1.675
LPC (O-36:4)	349	285	427	289	243	365	1.21	0.169	0.704	125.008	768.636	1.674
LPC (O-36:5)	8846	6228	13273	11118	8417	14358	0.80	0.272	0.733	125.008	766.612	1.674
LPC (O-38:1)	9041	8482	11865	8765	7909	10913	1.03	0.632	0.888	125.008	802.652	1.713
LPC (O-38:2)	3584	2866	3873	3075	2768	3513	1.17	0.272	0.733	125.006	800.683	1.713
LPC (O-38:3)	266	164	308	282	230	311	0.95	0.699	0.902	125.006	798.637	1.713
LPC (O-38:5)	5282	3332	5714	2057	0	4590	2.57	0.051	0.497	125.008	794.633	1.712
LPC (O-38:6)	519	450	586	509	428	612	1.02	0.938	0.959	125.006	792.635	1.712
LPC (O-40:2)	115668	97765	136778	106871	99451	126025	1.08	0.588	0.879	125.007	828.678	1.735

Lipid feature annotation	Carriers of pathogenic *LDLR* variant (n = 17)			Carriers with no detected pathogenic *LDLR* variant (n = 24)			Fold change	*p*-Value		Lipid annotation		
	Median	Q1	Q3	Median	Q1	Q3		Raw *p*-Value	FDR-adjusted *p*-Value	Scan m/z	m/z	Correction
LPC (O-40:3)	2296	1820	2775	2377	2093	2841	0.97	0.300	0.733	125.008	826.676	1.717
LPC (O-40:4)	880	815	1084	912	768	1071	0.96	0.769	0.910	125.007	824.674	1.735
LPC (O-40:5)	590	553	732	655	583	765	0.90	0.315	0.733	125.006	822.658	1.735
LPC (O-40:6)	7910	6083	10301	7871	6803	9662	1.00	0.688	0.902	125.008	820.666	1.735
Dimethylphosphatidylethanolamine (DMPE)												
DMPE (38:5)	18392	17198	20501	15552	12731	18234	1.18	0.061	0.497	625.558	766.612	1.659
Phosphatidylethanolamine (PE)												
PE (36:1)	21575	14876	23641	22826	19763	26969	0.95	0.345	0.778	605.583	746.634	1.623
PE (36:2)	824	710	975	745	571	944	1.11	0.307	0.733	603.575	744.629	1.623
PE (36:3)	858	626	1049	848	709	1052	1.01	0.621	0.888	607.573	748.607	1.623
PE (38:3)	13175	11243	16408	14358	12655	16906	0.92	0.286	0.733	629.582	770.650	1.659
PE (38:4)	7800	6788	8811	7581	5093	9032	1.03	0.745	0.910	627.576	768.636	1.659
PE (38:5)	4272	1384	5114	2207	0	4947	1.94	0.409	0.781	625.558	766.612	1.659
PE (40:6)	904	646	1008	958	840	1154	0.94	0.183	0.704	651.577	792.635	1.696

The data are presented as median relative signal abundance, along with the first (Q1) and third (Q3) quartiles. The fold change is the ratio of the median abundance of participants with a pathogenic *LDLR* variant to the median abundance of participants without a detected pathogenic *LDLR* variant. Raw *p*-Values were obtained using two-sided Mann-Whitney U tests. False discovery rate (FDR)-adjusted *p*-Values were calculated across all 142 positively ionized glycerophospholipid analytical features tested. Rows with the same lipid annotation that are distinguished by superscript letters (a, b, or c) represent different analytical feature records with distinct diagnostic scan m/z values. These rows should not be interpreted as representing independently identified lipid molecular species.

LipidView software, version 1.2: "Scan m/z" denotes the m/z of the diagnostic scan channel used for the analytical feature record. "Target m/z" denotes the m/z of the assigned precursor/molecular ion. "Correction" denotes the LipidView isotope correction factor used to correct for isotopic peak distribution. FDR = false discovery rate, LPC = lysophosphatidylcholine, and PC = phosphatidylcholine.
