# Supplementary material for: Plasma Lipidomic Phenotype Associated with Pathogenic LDLR Variants in Familial Hypercholesterolemia
Source: Int J Mol Sci. 2026 Oct 11;27(20):9011. doi: 10.3390/ijms27209011 (PMC13705200; doi:10.3390/ijms27209011)
Supplement: Supplementary file 1 [file ijms-27-09011-s001.zip › Table S4.pdf]

Table S4. Comparison of fatty acyl relative abundances detected in negative ionization mode according to pathogenic *LDLR* variant status.

Lipid species	Carriers of pathogenic LDLR variant (n = 17)			Carriers with no detected pathogenic LDLR variant (n = 24)			Fold change	*p*-Value		Lipid annotation		
	Median	Q1	Q3	Median	Q1	Q3		Raw *p*-Value	FDR-adjusted *p*-Value	Scan m/z	m/z	Correction
O-acyl-ω-hydroxy fatty acids (OAHFA)												
OAHFA (16:0/18:1)	206	108	319	501	225	753	0.41	0.012	0.053	255.243	535.385	1.480
OAHFA (16:0/18:2)	152	100	200	231	138	361	0.66	0.019	0.053	255.242	533.383	1.480
OAHFA (18:0/18:1)	130	88	234	247	158	390	0.53	0.027	0.053	283.275	563.413	1.514
OAHFA (18:1/18:1)	479	269	1,008	1,477	495	2,236	0.32	0.029	0.053	281.260	561.435	1.513
OAHFA (18:1/18:2)	157	112	288	416	159	523	0.38	0.024	0.053	281.258	559.362	1.513
OAHFA (18:1/20:2)	64	32	105	136	67	174	0.47	0.018	0.053	281.258	587.437	1.548
OAHFA (18:2/18:1)	174	93	253	319	155	649	0.55	0.047	0.069	279.245	559.362	1.000
OAHFA (18:2/18:2)	72	47	114	111	73	186	0.65	0.050	0.069	279.242	557.358	1.000
OAHFA (16:1/18:1)	46	25	79	101	40	172	0.46	0.063	0.077	253.227	533.383	1.480
OAHFA (18:2/28:1)	116	86	189	151	117	181	0.76	0.235	0.259	279.243	699.560	1.000
OAHFA (18:2/30:1)	208	136	303	244	189	285	0.85	0.386	0.386	279.244	727.583	1.000

All 11 negatively ionized fatty acyl analytical features are presented. Eight of these features showed nominally significant differences between groups in a two-sided Mann–Whitney U test ($p < 0.05$). OAHFA (18:2/18:2) was reported as $p = 0.050$ after rounding. The data are presented as median relative abundance, along with the first (Q1) and third (Q3) quartiles. The fold change (FC) was calculated as the ratio of the median abundance in participants with a pathogenic *LDLR* variant to the median abundance in participants without a detected pathogenic LDLR variant. Raw *p*-Values were obtained using two-sided Mann–Whitney U tests. False discovery rate (FDR)-adjusted *p*-Values were calculated across all 11 negatively ionized fatty acyl species tested.

LipidView software, version 1.2, was used. Fields: "Scan m/z" denotes the m/z of the diagnostic scan channel used for the analytical feature. "Target m/z" denotes the m/z of the assigned precursor or molecular ion. "Correction" denotes the LipidView isotope correction factor used to correct for isotopic peak distribution. FDR = false discovery rate, and OAHFA = O-acyl-ω-hydroxy fatty acid.
