# Supplementary material for: Plasma Lipidomic Phenotype Associated with Pathogenic LDLR Variants in Familial Hypercholesterolemia
Source: Int J Mol Sci. 2026 Oct 11;27(20):9011. doi: 10.3390/ijms27209011 (PMC13705200; doi:10.3390/ijms27209011)
Supplement: Supplementary file 1 [file ijms-27-09011-s001.zip › Table S4a.pdf]

Table S4a. Sensitivity analysis of fatty-acyl relative abundances detected in negative ionization mode according to pathogenic *LDLR* variant status after exclusion of two subsequently enrolled related participants.

**Lipid species**	**Carriers of pathogenic LDLR variant (n = 15)**			**Carriers with no detected pathogenic LDLR variant (n = 24)**			**Fold change**	***One – sided p-value Carriers LDLR < No detected LDLR***		***Two-sided p-Value Carriers LDLR ≠ No detected LDLR***		**Lipid annotation**		
	**Median**	**Q1**	**Q3**	**Median**	**Q1**	**Q3**		**Raw *p*-Value**	FDR-adjusted p-Value	**Raw *p*-Value**	FDR-adjusted p-Value	Scan m/z	Target m/z	Correction
								O-acyl-ω-hydroxy fatty acids (OAHFA)						
**OAHFA (16:0/18:2)**	152	100	195	231	138	361	0.66	0.010	0.040	0.019	0.080	255.242	533.383	1.480
**OAHFA (16:0/18:1)**	206	144	319	501	225	753	0.41	0.012	0.040	0.023	0.080	255.243	535.385	1.480
**OAHFA (18:1/ 20:2)**	64	42	105	136	67	174	0.47	0.014	0.040	0.028	0.080	281.258	587.437	1.548
**OAHFA (18:1/ 18:2)**	157	113	288	416	159	523	0.38	0.017	0.040	0.034	0.080	281.258	559.361	1.513
**OAHFA (18:1/ 18:1)**	479	411	1008	1477	495	2236	0.32	0.022	0.040	0.043	0.080	281.260	561.435	1.513
**OAHFA (18:0/ 18:1)**	130	94	234	247	158	390	0.53	0.022	0.040	0.043	0.080	283.275	563.413	1.514
**OAHFA (18:2/ 18:2)**	72	48	105	111	73	186	0.65	0.033	0.045	0.065	0.090	279.242	557.358	1.000
**OAHFA (18:2/ 18:1)**	174	104	230	319	155	649	0.55	0.033	0.045	0.065	0.090	279.245	559.361	1.000
**OAHFA (16:1/ 18:1)**	60	37	79	101	40	172	0.60	0.064	0.078	0.128	0.156	253.227	533.383	1.480
**OAHFA (18:2/ 28:1)**	111	86	146	151	117	181	0.74	0.078	0.086	0.156	0.172	279.243	699.560	1.581
**OAHFA (18:2/ 30:1)**	199	136	284	244	189	285	0.82	0.128	0.128	0.257	0.257	279.243	727.583	1.000

A sensitivity analysis was performed after excluding two related participants from the same families who were subsequently enrolled, leaving 15 participants with pathogenic *LDLR* variants and 24 participants without detected pathogenic *LDLR* variants. All 11 negatively ionized fatty-acyl analytical features are presented. Six features showed nominal between-group differences in the raw two-sided Mann–Whitney U test ($p < 0.05$). Data are presented as median relative abundance with the first (Q1) and third (Q3) quartiles. Fold change (FC) was calculated as the ratio of median abundance in pathogenic *LDLR* variant carriers to median abundance in participants without a detected pathogenic *LDLR* variant. False discovery rate (FDR)-adjusted *p*-Values were calculated across all 11 negatively ionized fatty-acyl features using the Benjamini–Hochberg procedure.

LipidView software, version 1.2, was used. “Scan m/z” denotes the m/z of the diagnostic scan channel used for the analytical feature. “Target m/z” denotes the m/z of the assigned precursor/molecular ion. “Correction” denotes the LipidView isotope correction factor used to correct for isotopic peak distribution. FDR = false discovery rate; OAHFA = O-acyl-ω-hydroxy fatty acid.
