# Supplementary material for: Plasma Lipidomic Phenotype Associated with Pathogenic LDLR Variants in Familial Hypercholesterolemia
Source: Int J Mol Sci. 2026 Oct 11;27(20):9011. doi: 10.3390/ijms27209011 (PMC13705200; doi:10.3390/ijms27209011)
Supplement: Supplementary file 1 [file ijms-27-09011-s001.zip › Table S5.pdf]

Table S5. Comparison of sphingolipid relative abundances detected in positive ionization mode according to pathogenic *LDLR* variant status.

Lipid species	Carriers of pathogenic *LDLR* variant (n = 17)			Carriers with no detected pathogenic LDLR variant (n = 24)			Fold change	*p*-Value		Lipid annotation		
	Median	Q1	Q3	Median	Q1	Q3		Raw *p*-Value	FDR-adjusted *p*-Value	Scan m/z	m/z	Correction
				Sphingomyelin (SM)								
SM (36:3)	295,421	211,807	364,685	376,634	288,242	401,303	0.78	0.025	0.535	184.086	743.602	1.625
SM (40:0)	4,300	141	5,065	676	0	3,755	6.36	0.049	0.535	184.086	789.669	1.697
SM (40:3)	234,227	218,601	269,371	190,898	164,375	228,326	1.23	0.049	0.535	184.087	783.626	1.696
SM (42:3)	184,827	164,131	273,322	155,987	125,254	182,977	1.18	0.029	0.535	184.087	811.658	1.735
SM (42:4)	67,945	47,017	74,420	51,402	43,706	60,211	1.32	0.037	0.535	184.086	809.639	1.734
SM (30:3/4)	13382	9381	17232	12974	11488	14573	1.03	1.000	1.000	184.086	675.517	1.523
SM (32:2/3)	7987	4616	10723	6970	5045	8018	1.15	0.486	0.936	184.086	689.565	1.554
SM (32:2/4)	20060	13265	22778	17525	13851	21107	1.14	0.486	0.936	184.086	705.553	1.558
SM (32:3/4)	216362	142207	250894	202677	159139	228227	1.07	0.588	0.936	184.087	703.601	1.557
SM (32:4/4)	23118	19263	25949	20719	18083	24855	1.12	0.632	0.936	184.086	701.588	1.557
SM (34:1/4)	8114	6672	9640	7725	6505	9292	1.05	0.506	0.936	184.086	735.609	1.593
SM (34:2/3)	4138	155	6690	1880	0	4775	2.20	0.197	0.932	184.085	717.592	1.589
SM (34:2/4)	13271	10652	17490	12309	10799	14885	1.08	0.841	0.987	282.297	733.589	1.593
SM (34:3/4)	37177	31049	55307	35936	31486	43857	1.03	0.546	0.936	184.086	731.583	1.593
SM (34:4/4)	16833	12757	23983	14411	12215	18135	1.17	0.178	0.932	184.086	729.555	1.592
SM (36:1/3)	5905	3779	7887	5018	3303	5749	1.18	0.240	0.932	184.086	747.597	1.626
SM (36:1/4)	6588	5910	7383	6457	5517	7708	1.02	0.865	0.994	184.085	763.645	1.630
SM (36:2/3)	8157	6653	10624	8164	6028	10683	1.00	0.793	0.973	184.086	745.618	1.625
SM (36:2/4)	192204	178365	221292	194215	165191	225799	0.99	0.963	1.000	184.087	761.640	1.629
SM (36:3/3)	4809	979	6008	1952	0	3692	2.46	0.156	0.932	184.086	743.617	1.625
SM (36:4/4)	18441	16342	20020	17764	15094	19108	1.04	0.449	0.936	184.086	757.606	1.629
SM (38:0/4)	7072	6074	7741	6621	4285	7928	1.07	0.676	0.936	184.086	793.610	1.667
SM (38:1/3)	7598	5523	9117	7442	6495	9506	1.02	0.654	0.936	184.086	775.626	1.663
SM (38:1/4)	888	0	2395	588	0	1938	1.51	0.733	0.958	184.084	791.627	1.667
SM (38:2/3)	13518	9828	17615	14065	10814	16613	0.96	0.938	0.994	184.086	773.647	1.662
SM (38:2/4)	38938	32017	44942	36058	29801	40159	1.08	0.632	0.936	266.284	789.664	1.666
SM (38:3/3)	6861	6199	8406	7521	5608	8875	0.91	0.546	0.936	184.086	771.651	1.662
SM (38:3/4)	206012	189007	269644	222548	195898	276434	0.93	0.430	0.936	184.087	787.657	1.666
SM (38:4/3)	14400	11884	17678	13412	10269	18342	1.07	0.654	0.936	184.086	769.645	1.662
SM (38:4/4)	132173	113616	161006	134618	120478	158526	0.98	0.769	0.966	184.087	785.638	1.666
SM (40:0/3)	0	0	1106	0	0	0	NC	0.099	0.760	184.085	805.655	1.701
SM (40:1/3)	3476	1010	5851	3308	1663	5521	1.05	0.745	0.958	184.086	803.653	1.701
SM (40:1/4)	0	0	2487	0	0	1153	NC	0.257	0.932	184.085	819.651	1.705
SM (40:2/3)	20039	14558	28509	22580	18005	26132	0.89	0.817	0.980	184.086	801.619	1.700

Lipid species	Carriers of pathogenic *LDLR* variant (n = 17)			Carriers with no detected pathogenic LDLR variant (n = 24)			Fold change	*p*-Value		Lipid annotation		
	Median	Q1	Q3	Median	Q1	Q3		Raw *p*-Value	FDR-adjusted *p*-Value	Scan m/z	m/z	Correction
SM (40:2/4)	5211	1544	6321	4626	3441	5788	1.13	0.938	0.994	184.086	817.652	1.704
SM (40:3/3)	23281	19676	31714	24649	20283	29284	0.94	0.914	0.994	184.086	799.617	1.700
SM (40:3/4)	43648	28843	55425	43468	35025	52771	1.00	0.988	1.000	184.087	815.692	1.704
SM (40:4/2)	16902	14450	21171	20417	15807	29646	0.83	0.330	0.936	184.086	781.607	1.696
SM (40:4/3)	16545	12807	20480	15366	11851	16385	1.08	0.134	0.901	184.086	797.648	1.700
SM (40:4/4)	144799	119395	180314	152953	126959	173159	0.95	0.914	0.994	184.087	813.676	1.704
SM (42:0/3)	4876	129	5732	1340	0	3762	3.64	0.085	0.760	184.085	833.683	1.740
SM (42:2/3)	1122	0	6388	1543	0	3270	0.73	0.402	0.936	184.086	829.679	1.739
SM (42:3/3)	8303	6679	13764	8691	7071	10193	0.96	0.430	0.936	184.086	827.630	1.739
SM (42:4/3)	4577	149	6539	2643	1560	5251	1.73	0.411	0.936	184.085	825.675	1.738
SM (44:0/4)	0	0	0	0	0	0	NC	0.579	0.936	184.086	877.737	1.784
SM (44:3/2)	2320	0	4399	1225	0	1663	1.89	0.283	0.936	184.084	839.689	1.774
SM (44:4/2)	7769	6908	9610	7049	6197	8274	1.10	0.234	0.932	266.270	837.687	1.774
Dihexosylceramide (Hex2Cer)												
Hex2Cer (26:0/4)	142	113	237	160	125	250	0.89	0.676	0.936	264.279	784.505	1.595
Hex2Cer (28:0/4)	186	144	263	216	159	287	0.86	0.421	0.936	264.279	812.537	1.631
Gangliosides (GD)												
GD1 (28:2/4)	388	0	598	69	0	274	5.60	0.259	0.932	266.270	878.418	2.627
GD1 (28:3/4)	0	0	0	0	0	0	NC	0.647	0.936	266.270	877.410	2.627
GD2 (30:0/4)	235	201	292	249	187	298	0.94	0.676	0.936	264.279	813.423	2.484
GM1 (26:1/2)	444	324	520	425	347	479	1.04	0.654	0.936	264.279	703.867	2.210
GD3 (26:2/2)	211	137	254	193	163	243	1.10	0.734	0.958	264.279	811.389	2.460

The results show all 54 positively ionized sphingolipid species that were analyzed. The results revealed statistically significant differences in the raw test (two-sided Mann-Whitney U test, $p < 0.05$). NC indicates that the fold change could not be calculated because the median abundance in the panel-negative group was zero (the denominator). The data are presented as median relative abundance, along with the first (Q1) and third (Q3) quartiles. The fold change was calculated as the ratio of the median abundance in participants with a pathogenic *LDLR* variant to the median abundance in participants with no detected pathogenic *LDLR* variant. Raw *p*-Values were obtained using two-sided Mann-Whitney U tests. False discovery rate (FDR)-adjusted *p*-Values were calculated for all 54 positively ionized sphingolipid species. LipidView software, version 1.2, was used. "Scan m/z" denotes the m/z of the characteristic diagnostic scan channel used for the analytical feature. "Target m/z" denotes the m/z of the assigned precursor/molecular ion. "Correction" denotes the LipidView isotope correction factor used to correct for isotopic peak distribution. FDR = false discovery rate; SM = sphingomyelin; Hex2Cer = dihexosylceramide; GM1, GD1, GD2, and GD3 = gangliosides.
