# Supplementary material for: Plasma Lipidomic Phenotype Associated with Pathogenic LDLR Variants in Familial Hypercholesterolemia
Source: Int J Mol Sci. 2026 Oct 11;27(20):9011. doi: 10.3390/ijms27209011 (PMC13705200; doi:10.3390/ijms27209011)
Supplement: Supplementary file 1 [file ijms-27-09011-s001.zip › Table S6.pdf]

Table S6. Comparison of lipid relative abundances showing nominal between-group differences in glycerolipids and sterols detected in positive ionization mode and sphingolipids detected in negative ionization mode according to pathogenic *LDLR* variant status.

Lipid species	Carriers of pathogenic *LDLR* variant (n = 17)			Carriers with no detected pathogenic *LDLR* variant (n = 24)			Fold change	*p*-Value		Lipid annotation		
	**Median**	**Q1**	**Q3**	**Median**	**Q1**	**Q3**		**Raw *p*-Value**	**FDR-adjusted *p*-Value**	**Scan m/z**	**Target m/z**	**Correction**
				Glycerolipids (GL)								
TAG (54:6/22:6)	119	57	247	299	195	404	0.40	0.027	0.680	551.503	896.770	1.925
TAG (38:3/14:0)	150	66	232	113	10	243	1.33	0.378	0.676	433.331	678.567	1.609
TAG (38:3/18:0)	841	283	1366	559	22	1416	1.50	0.448	0.685	377.269	678.567	1.609
TAG (46:7/19:0)	590	403	662	483	400	656	1.22	0.889	0.962	467.316	782.629	1.759
TAG (54:5/16:0)	426	252	1041	889	494	1324	0.48	0.188	0.486	625.519	898.786	1.926
TAG (54:5/18:1)	2897	1969	5106	3961	2504	4977	0.73	0.467	0.685	599.503	898.786	1.926
TAG (54:5/18:2)	2406	1683	3948	3449	1828	4640	0.70	0.412	0.685	601.519	898.786	1.926
TAG (54:6/16:0)	235	178	573	321	193	675	0.73	0.665	0.758	623.503	896.770	1.926
TAG (54:6/18:1)	722	423	1055	486	380	750	1.49	0.204	0.497	597.488	896.770	1.926
TAG (54:6/18:2)	1137	574	1888	852	612	1215	1.33	0.265	0.528	599.503	896.770	1.926
TAG (54:6/18:3)	712	422	939	480	321	617	1.48	0.154	0.456	601.519	896.770	1.926
TAG (54:7/18:2)	266	178	478	297	174	447	0.90	0.769	0.892	597.488	894.755	1.925
TAG (54:7/18:3)	238	150	356	273	149	377	0.87	0.588	0.758	599.503	894.755	1.925
TAG (54:7/20:4)	178	136	262	168	121	245	1.06	0.448	0.685	573.488	894.755	1.925
TAG (54:7/22:6)	210	117	435	441	205	670	0.48	0.070	0.456	549.488	894.755	1.925
TAG (56:6/16:0)	273	0	688	389	0	647	0.70	0.580	0.737	651.535	924.802	1.969
TAG (56:6/18:1)	570	0	1919	705	0	1254	0.81	0.913	0.962	625.519	924.802	1.969
TAG (56:6/18:2)	164	0	497	218	104	381	0.75	0.975	0.975	627.535	924.802	1.969
TAG (56:6/20:3)	176	90	373	209	71	256	0.84	0.419	0.685	601.519	924.802	1.969
TAG (56:7/16:0)	509	263	860	841	428	1174	0.61	0.089	0.456	649.519	922.786	1.969
TAG (56:7/18:1)	850	452	1054	889	432	1278	0.96	0.546	0.699	623.503	922.786	1.969
TAG (56:7/18:2)	459	364	629	437	349	635	1.05	0.486	0.688	625.519	922.786	1.969
TAG (56:7/20:4)	779	617	1331	647	443	975	1.20	0.189	0.497	601.519	922.786	1.969
TAG (56:8/16:0)	107	77	218	242	108	358	0.44	0.066	0.456	647.503	920.770	1.968
TAG (56:8/18:2)	238	163	316	310	145	460	0.77	0.361	0.676	623.503	920.770	1.968
TAG (56:8/20:4)	327	209	456	208	152	323	1.57	0.092	0.456	599.503	920.770	1.968
TAG (56:8/20:5)	186	162	252	174	99	324	1.07	0.963	0.970	601.519	920.770	1.968
TAG (56:9/22:6)	113	63	173	156	68	239	0.73	0.228	0.516	573.488	918.755	1.968
TAG (58:8/18:1)	101	0	389	121	0	158	0.84	0.761	0.892	649.519	948.802	2.013
TAG (58:9/18:1)	88	67	198	110	46	164	0.80	0.853	0.892	647.503	946.786	2.013
TAG (58:9/22:6)	268	171	407	388	148	525	0.69	0.449	0.685	601.519	946.786	2.013
TAG (58:10/22:6)	133	73	151	149	73	216	0.89	0.395	0.685	599.503	944.770	2.012
DGDG (30:5/18:2)	0	0	0	0	0	4837	NC	0.062	0.456	575.270	872.537	1.719
DGDG (30:5/18:3)	0	0	0	0	0	890	NC	0.056	0.456	577.286	872.537	1.719

Lipid species	Carriers of pathogenic *LDLR* variant (n = 17)			Carriers with no detected pathogenic *LDLR* variant (n = 24)			Fold change	*p*-Value		Lipid annotation		
	Median	Q1	Q3	Median	Q1	Q3		Raw *p*-Value	FDR-adjusted *p*-Value	Scan m/z	Target m/z	Correction
DGDG (32:6/18:3)	1217	563	1562	1227	794	1510	0.99	0.889	0.962	603.301	898.552	1.758
DGDG (32:6/20:3)	318	223	794	544	311	656	0.59	0.506	0.688	575.270	898.552	1.758
DGDG (32:6/20:4)	1614	698	3234	2530	1311	3258	0.64	0.477	0.688	577.286	898.552	1.758
DGDG (32:7/20:4)	819	499	1341	555	498	1097	1.48	0.353	0.660	575.270	896.537	1.758
DGDG (32:7/20:5)	274	188	615	386	206	633	0.71	0.733	0.838	577.286	896.537	1.758
DGDG (32:8/20:5)	216	183	389	317	204	478	0.68	0.330	0.595	575.270	894.521	1.757
DGDG (34:7/20:4)	626	0	2128	777	0	1434	0.81	0.912	0.962	603.301	924.568	1.798
DGDG (34:7/22:6)	85	52	290	190	92	287	0.45	0.118	0.456	579.301	924.568	1.798
DGDG (34:8/20:5)	331	258	510	307	183	580	1.08	0.769	0.892	603.301	922.552	1.797
DGDG (34:8/22:6)	1298	625	2699	2299	1140	3805	0.56	0.104	0.456	577.286	922.552	1.797
DGDG (34:9/22:6)	696	299	1161	1011	400	1631	0.69	0.159	0.486	575.270	920.537	1.797
DGDG (36:9/22:6)	151	0	729	231	0	387	0.65	0.936	0.962	603.301	948.568	1.838
MADAG (42:6/22:6)	369	0	452	66	0	396	5.56	0.287	0.595	369.336	714.603	1.678
MADAG (46:1/19:0)	665	523	1020	800	686	1016	0.83	0.134	0.456	465.430	780.744	1.758
MADAG (54:6/17:2)	153	65	177	169	84	264	0.91	0.286	0.592	599.540	882.791	1.921
MADAG (54:11/16:0)	0	0	0	0	0	1129	NC	0.108	0.456	599.446	872.713	1.919
MADAG (54:11/16:1)	0	0	0	0	0	0	NC	0.170	0.456	601.462	872.713	1.919
MADAG (54:11/18:1)	0	0	0	0	0	2142	NC	0.056	0.492	573.430	872.713	1.919
MADAG (54:11/20:4)	0	0	0	0	0	201	NC	0.062	0.456	551.446	872.713	1.919
MADAG (54:12/16:0)	0	0	0	0	0	309	NC	0.108	0.975	597.430	870.697	1.919
MADAG (54:12/16:1)	0	0	0	0	0	437	NC	0.129	0.456	599.446	870.697	1.919
MADAG (54:12/18:1)	0	0	0	0	0	211	NC	0.099	0.456	571.415	870.697	1.919
MADAG (54:12/18:2)	0	0	0	0	0	0	NC	0.219	0.456	573.430	870.697	1.919
MADAG (54:12/18:3)	0	0	0	0	0	577	NC	0.118	0.528	575.446	870.697	1.919
Sterols (ST)												
CE (20:4)	12,850	8,851	18,436	9,530	6,977	12,086	1.35	0.027	0.378	369.377	690.567	1.703
CE (16:0)	2646	779	3785	3015	1889	3498	0.88	0.506	0.870	369.377	642.567	1.630
CE (16:1)	1056	844	1778	1263	1040	1692	0.84	0.199	0.870	369.377	640.551	1.629
CE (18:0)	432	258	522	412	356	502	1.05	0.486	0.870	369.377	670.598	1.667
CE (18:1)	11744	7231	13627	11144	10241	13025	1.05	0.745	0.870	369.377	668.583	1.666
CE (18:2)	41143	28415	45714	38667	35499	51715	1.06	0.486	0.870	369.377	666.567	1.666
CE (18:3)	1503	1161	1640	1323	1130	1647	1.14	0.654	0.870	369.377	664.551	1.666
CE (20:0)	407	228	609	814	347	1078	0.50	0.104	0.731	369.377	698.630	1.705
CE (20:3)	1081	879	1236	1065	816	1177	1.01	0.526	0.870	369.377	692.583	1.704
CE (20:5)	1305	915	1890	1469	928	2629	0.89	0.526	0.870	369.377	688.551	1.703
CE (22:2)	441	284	946	512	247	797	0.86	0.938	1.000	369.377	722.630	1.743

Lipid species	Carriers of pathogenic *LDLR* variant (n = 17)			Carriers with no detected pathogenic *LDLR* variant (n = 24)			Fold change	*p*-Value		Lipid annotation		
	Median	Q1	Q3	Median	Q1	Q3		Raw *p*-Value	FDR-adjusted *p*-Value	Scan m/z	Target m/z	Correction
CE (22:3)	0	0	70	0	0	104	NC	1.000	1.000	369.377	720.614	1.742
CE (22:6)	1210	864	1359	1266	899	1471	0.96	0.676	0.870	369.377	714.567	1.741
CE (24:0)	15	0	57	22	0	100	0.69	0.708	0.870	369.377	754.692	1.783
				Sphingolipids (SP)								
SM (32:2)	563	459	596	482	446	508	1.17	0.048	0.348	168.048	763.559	1.558
SM (32:3)	3,658	3,010	3,815	3,038	2,677	3,311	1.20	0.027	0.348	168.049	761.583	1.557
SM (28:4;3)	288	208	329	258	232	285	1.12	0.305	0.640	168.049	687.436	1.485
SM (30:0;4)	257	149	432	296	143	351	0.87	0.608	0.760	168.049	739.524	1.524
SM (30:1;4)	668	329	910	706	314	767	0.95	0.898	0.945	168.049	737.509	1.524
SM (30:3;4)	430	311	484	369	320	430	1.17	0.360	0.655	168.049	733.477	1.523
SM (32:2;3)	407	306	502	369	338	411	1.10	0.248	0.620	168.049	747.529	1.555
SM (32:4;4)	520	397	632	480	414	518	1.08	0.188	0.620	168.049	759.493	1.558
SM (34:3;4)	576	488	737	526	452	653	1.10	0.223	0.620	168.049	789.540	1.594
SM (34:4;4)	317	244	383	277	221	318	1.15	0.052	0.348	168.049	787.524	1.593
SM (36:2;4)	361	341	395	365	353	401	0.99	0.511	0.760	168.049	819.587	1.630
SM (36:3;4)	669	618	714	690	598	739	0.97	0.773	0.859	168.049	817.571	1.630
SM (38:2;4)	285	208	381	268	224	333	1.06	0.597	0.760	168.049	847.618	1.667
SM (38:3;4)	661	583	705	694	592	735	0.95	0.597	0.760	168.049	845.603	1.667
SM (38:4;4)	470	405	504	453	395	512	1.04	1.000	1.000	168.049	843.587	1.667
SM (40:3;2)	262	234	324	234	194	290	1.12	0.102	0.509	168.049	841.644	1.697
SM (40:3;4)	317	252	349	318	247	346	1.00	0.724	0.852	168.049	873.634	1.705
SM (40:4;4)	839	715	898	732	624	845	1.15	0.223	0.620	168.049	871.618	1.705
SM (42:3;2)	439	397	514	440	314	469	1.00	0.320	0.640	168.049	869.675	1.735
SGalCer (42:3;2)	349	295	402	319	298	370	1.09	0.564	0.760	96.960	886.608	1.863

The results show all 58 glycerolipid and 14 sterol species detected in positive ionization mode, as well as all 20 sphingolipid species detected in negative ionization mode. Statistically significant differences were observed in the raw test (two-sided Mann-Whitney U test, $p < 0.05$). NC indicates that the fold change could not be calculated because the median abundance in the panel-negative group was zero (the denominator).

The data are presented as median relative abundance, along with the first (Q1) and third (Q3) quartiles. The fold change was calculated as the ratio of the median abundance in participants with a pathogenic *LDLR* variant to the median abundance in participants without a detected pathogenic *LDLR* variant. Raw *p*-Values were obtained using two-sided Mann-Whitney U tests. False discovery rate (FDR)-adjusted *p*-Values were calculated across all species within the relevant lipid class: 58 glycerolipids (GL), 14 sterols, and 20 sphingolipids (SP).

LipidView software, version 1.2, was used. Fields: Scan m/z denotes the m/z of the characteristic diagnostic scan channel used for the analytical feature. "Target m/z" denotes the m/z of the assigned precursor/molecular ion. "Correction" denotes the LipidView isotope correction factor. FDR stands for false discovery rate. Other terms used in this study include TAG = triacylglycerol; DGDG = digalactosyldiacylglycerol; MADAG = monoalkyl diacylglycerol; CE = cholesteryl ester; SM = sphingomyelin; SGalCer = sulfogalactosylceramide.
